# Insight into the factors controlling the equilibrium of allylic azides†

**DOI:** 10.1039/c9ra10093h

**Published:** 2020-01-27

**Authors:** Margarita M. Vallejos, Guillermo R. Labadie

**Affiliations:** Laboratorio de Química Orgánica, IQUIBA-NEA, Universidad Nacional del Nordeste, CONICET, FACENA Av. Libertad 5460 Corrientes 3400 Argentina vallejos.marga@gmail.com m.vallejos@conicet.gov.ar +54-379-4457996 ext. 104; Instituto de Química Rosario, UNR, CONICET Suipacha 531 S2002LRK Rosario Argentina; Departamento de Química Orgánica, Facultad de Ciencias Bioquímicas y Farmacéuticas, Universidad Nacional de Rosario Suipacha 531 S2002LRK Rosario Argentina

## Abstract

Several allylic azides with different double bond substitutions were studied to understand the factors, governing their equilibrium using density functional theory along with the quantum theory of atoms in molecules, non-covalent interactions and natural bond orbital approaches. The results showed that the hydroxyl group or heteroatoms in allylic azides interact with the molecule through an electrostatic weak interaction in each pair of regioisomers. The equilibrium shifts of substituted allylic azides, compared to non-substituted allylic azides, were not attributed to the presence of specific interactions, such as hydrogen bonds. The observed equilibrium shifts stemmed mainly from the strengthening and weakening of negative hyperconjugative interactions, which were affected by the weak interaction involving the proximal substituent in each regioisomer. A good linear correlation was obtained between the hyperconjugative energies of πC=C→σ*Z_b_ interactions and the calculated percentages of the secondary azide and tertiary azide in the equilibrium mixture. Also, the effect of the aromatic ring substituent was analysed using such approaches. This study not only provides insights into the factors controlling the stabilities of the substituted allylic azides, but also settles the basis to predict the regioisomer predominance in the equilibrium mixture.

## Abstract 2

The factors controlling the allyl azides equilibrium has been studied by different theoretical approaches setting the basis to predict the regioisomers predominance in the equilibrium mixture.

## Graphical abstract

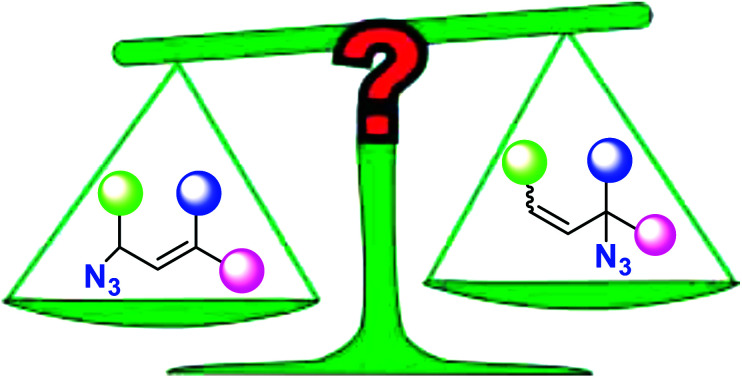

## Introduction

Organic azides are versatile substrates for use in reactions such us the Staudinger reaction, Schmidt reaction and Curtius rearrangement.^1–3^ Also, since the introduction of copper(i) azide–alkyne cycloaddition, thousands of compounds have been prepared.^4–6^ Azide bioorthogonality has promoted this functional group's introduction on metabolites and proteins in many different studies.^7–10^

**Fig. 1:**
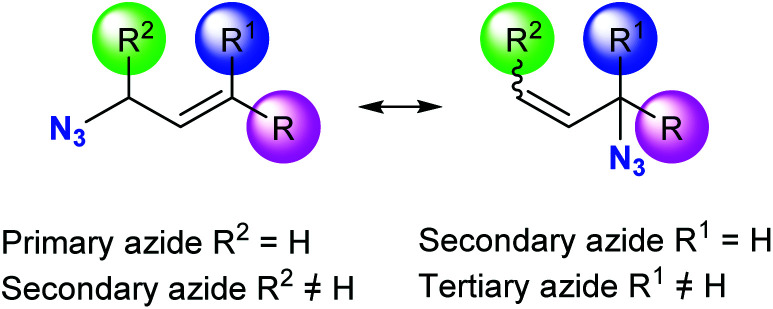
[3,3]-Sigmatropic rearrangement of allyl azides.

Allylic azides are the building blocks for the synthesis of many natural products and nitrogen-containing heterocycles of pharmacological relevance.^11–14^ Despite the importance of these useful synthons, their applicability in synthetic schemes has been difficult due to their existence as regioisomeric mixtures that interconvert rapidly at room temperature, thus being, in general, inseparable (Fig. 1).^15,16^ The allylic azide rearrangement was first reported by Gagneux, Winstein and Young in 1960 (known as Winstein's rearrangement).^15^ Lately, Vanderwerf and Heasley^17^ found that tertiary and secondary allylic azides rearrange faster than the primary ones existing in the equilibrium mixture, leading to a predominant population of the latter.

Allylic azide rearrangements are controlled by thermodynamic stabilization of the more substituted C=C double bond. Also, the distribution of the regioisomers is affected by the steric bulkiness and the conjugations of the double bonds. It has widely been assumed that the rearrangement of the allylic azides occurs through a concerted [3,3]-sigmatropic mechanism *via* a cyclic transition structure.^18,19^ However, an ionic mechanism was recently proposed for the rearrangement of allylic azides at high temperatures or under Lewis acidic conditions.^20^

**Scheme 1:**
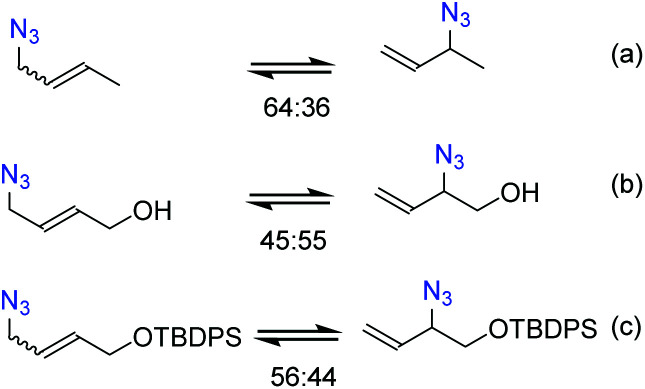
Equilibrium distribution of (a) crotyl azide, (b) hydroxyl-crotyl azide and, (c) its silyl-protected analogue.

It is necessary to control the thermodynamic ratio of the regioisomeric azides, which generally depends on the substrate, in order to be synthetically useful.^21^ Different groups have studied the rearrangement of substituted allylic azides to understand the main factors governing the equilibrium. Sharpless and co-workers^22^ noted that the equilibrium of hydroxylated allyl azides was shifted towards secondary azides compared to the parent aliphatic azide (Scheme 1b). This shift was attributed to the formation of hydrogen bonds between the hydroxyl and azide groups.^22–24^ Recently, Topczewski and co-workers^25^ observed that a silyl-protected analogue of hydroxyl-crotyl azide also showed a similar shift towards branched regioisomers with the OR group close to the azide group (Scheme 1c).

The allylic azide equilibrium ratio might be also significantly biased by an aromatic ring substituent. Overall, only conjugated regioisomers with the aromatic ring have been evidenced.^26,27^ We previously performed a theoretical study using density functional theory and the quantum theory of atoms in molecules (QTAIM) approach^28,29^ to rationalize the experimental results. Those results showed a high dependency of the double bond substitution on the prenylazides with different chain lengths of the prenylazide. Topological analysis of the electron-charge density revealed that the effect of the aromatic substituent was strictly electronic, with scarce or null contribution from the steric factor.^30^

There are few computational studies on the allylic azide rearrangement and most of these have been based on the energetic and geometric parameters to analyse the effects that control the equilibrium.^23,31^ Herein, several representative allylic azides (alkyl, aryl and hydroxylated allylic azides) with different degrees of substitution on the double bond were chosen to study the effects of the substituent group on the stabilization of the regioisomers. Such effects were evaluated using the quantum topological methods by means of the QTAIM and non-covalent interactions (NCI) analyses and natural bond orbital (NBO) analysis.^32,33^ The obtained results provide a better understanding of the factors that govern the equilibrium, and might help predict the regioisomer composition of allylic azides in the equilibrium.

## Methodology

All the geometries were optimized with the M06-2X^34^ functional and the 6-31+G(d,p) basis set. Single-point energies were calculated with the larger 6-311++G(d,p) basis set and the implicit SMD^35^ solvation model with chloroform as the solvent. This level of theory for the calculation was chosen after an initial study for azides 1 and 2 using different DFT functionals (M06-2X,^34^ B3LYP^36,37^ and MPWB1K^38^) in conjugation with the 6-31+G(d) and 6-311++G(d,p) basis sets in the gas phase for the optimization and frequency calculation and 6-311++G(d,p) for the single-point calculation in chloroform (see ESI†). The reported free energies were calculated at 298.15 °K and 1 atm in chloroform.

As was established, the Winstein rearrangement is thermodynamically controlled, so this study focused on the regioisomers; however, for each reaction, the transition structure was localized (see ESI†). The calculated free energy barriers for the rearrangements of the azides under study were relatively low (<30 kcal mol^−1^), indicating that those could spontaneously occur at room temperature. Frequency calculations were computed to verify the nature of the stationary points as true minima or as first-order transition structures and to evaluate the thermal corrections. The intrinsic reaction coordinate (IRC) was further performed to check the energy profiles connecting each TS to the two associated minima.

Topological analyses were carried out with QTAIM^28^ and non-covalent interactions (NCI), using the AIMALL^39^ and NCIPLOT 3.0 (ref. 40) programs, respectively. Hyperconjugative interactions were evaluated using the Natural Bond Orbital program (NBO 3.1).^41^

## Results and discussion

The present study report is divided into three sections according to the types of allylic azides: primary *vs.* secondary, primary *vs.* tertiary and secondary *vs.* secondary. The percentage of *cis* isomer in the equilibrium mixture was generally low (or negligible) and therefore it was not computed for practicality.

### Primary *vs.* secondary azides

The relative Gibbs energies, and the experimental and calculated equilibrium ratio for the azides under study are summarized in Table 1.

**Table:** Relative free energies (Δ*G*, kcal mol^−1^) and calculated and experimental equilibrium ratios for the azides under study^a^

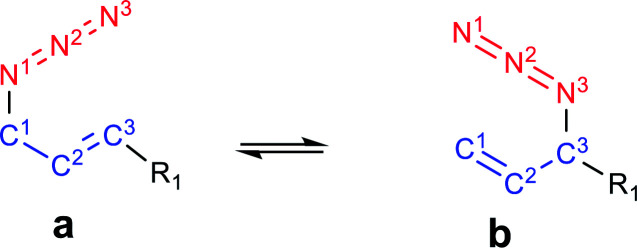				
Azide (*X*)	R_1_	Δ*G* (*X*_b_ − *X*_a_)	*X*_a_ : *X*_b_ ratio	
			Calcd^a^	Exp.^b^^,^^c^
1	Me	1.10	86 : 14	67 : 33 (ref. 22)
2	CH_2_OH	−0.02	49 : 51	45 : 55 (ref. 22)
3	OTMS	0.67	75 : 25	56 : 44 (ref. 25)
4	Ph	3.72	100 : 0	100 : 0 (ref. 30)

a Ratios were computed using Boltzmann factors based on Δ*G*.

b 1a (57% *E-trans*, 10% *Z-cis*).

c The experimental data for azide 3 correspond to hydroxyl-crotyl azide derivative –OTBDPS, structurally similar to –OTMS, 3a (52% *E-trans*, 4% *Z-cis*).

The primary azides 1a, 3a and 4a were more stable by 1.10, 0.67 and 3.72 kcal mol^−1^ than the corresponding secondary ones, being the calculated ratio *X*_a_ : *X*_b_ in agreement with the experimental results. Also, for azide 1, it was in accordance with the previous calculation at other levels of theory.^25,42^ The energy difference between 2a and 2b was lower, providing a ratio that slightly favoured the second. In some cases, the energy difference between both regioisomers was smaller than 1 kcal mol; however, the calculated ratios indicated a shift towards the secondary azides for 2 and 3 compared to 1, which correlated well with the observed equilibrium trend.

**Fig. 2:**
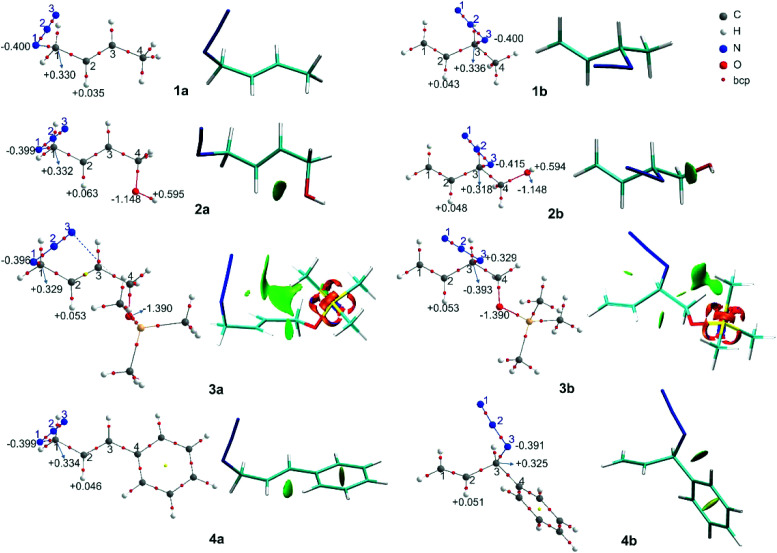
Molecular graphs of the azides 1–4 (left). For selected atoms, the atomic charges *q* (Ω) are given in *e*. NCI gradient isosurfaces (right), represented at an isovalue of 0.5 a.u. and blue-green-red color scale from −0.05 < sign(*λ*_2_)*ρ* < +0.05 a.u.

In azide 2b, the OH group is directed towards N^3^ (*d*_H⋯N_ = 2.38 Å; <svg xmlns="http://www.w3.org/2000/svg" version="1.0" width="10.400000pt" height="16.000000pt" viewBox="0 0 10.400000 16.000000" preserveAspectRatio="xMidYMid meet"><metadata> Created by potrace 1.16, written by Peter Selinger 2001-2019 </metadata><g transform="translate(1.000000,15.000000) scale(0.011667,-0.011667)" fill="currentColor" stroke="none"><path d="M80 1160 l0 -40 40 0 40 0 0 -40 0 -40 40 0 40 0 0 -40 0 -40 40 0 40 0 0 -40 0 -40 40 0 40 0 0 -40 0 -40 40 0 40 0 0 -40 0 -40 40 0 40 0 0 -40 0 -40 40 0 40 0 0 80 0 80 -40 0 -40 0 0 40 0 40 -40 0 -40 0 0 40 0 40 -40 0 -40 0 0 40 0 40 -40 0 -40 0 0 40 0 40 -40 0 -40 0 0 40 0 40 -80 0 -80 0 0 -40z M560 520 l0 -40 -40 0 -40 0 0 -40 0 -40 -40 0 -40 0 0 -40 0 -40 -40 0 -40 0 0 -40 0 -40 -40 0 -40 0 0 -40 0 -40 -40 0 -40 0 0 -40 0 -40 -40 0 -40 0 0 -40 0 -40 80 0 80 0 0 40 0 40 40 0 40 0 0 40 0 40 40 0 40 0 0 40 0 40 40 0 40 0 0 40 0 40 40 0 40 0 0 40 0 40 40 0 40 0 0 80 0 80 -40 0 -40 0 0 -40z"/></g></svg> OH–N^3^ = 107°), which could be a hydrogen bond or a coulombic interaction as was previously proposed to explain the equilibrium shift. To obtain more information about this interaction, a topological analysis of the electron density distribution based on the QTAIM was performed. This approach is one of the most popular for assessing whether a hydrogen bond is present. From this approach, the presence of a bond critical point, bcp (3,−1), between a hydrogen bond donor group and a hydrogen bond acceptor group along the bond path connecting two interacting atoms is considered to be a characteristic of hydrogen bonding.^43^ The molecular graphs of the azides are depicted in Fig. 2 (the topological properties evaluated at the bcps are listed in Table S2, in the ESI†).

According to the QTAIM analysis (Fig. 2), no bcp was observed between the hydrogen of the OH group and the N^3^ atom in 2b. On the basis of this theory, this may indicate that there was no hydrogen bond interaction in the 2b regioisomer. However, there are some criticisms of the interpretation of QTAIM theory, particularly for weak long-range bonds like hydrogen bonds.^44^

The NCI index is based on the electron density and its derivatives and this approach allows the identification of non-covalent weak interactions in a molecular system, particularly those interactions that cannot be revealed from an analysis of the density values.^45^ The NCI plot enables the visualization through the space of the gradient isosurfaces and provides qualitative information on the interacting molecular regions.

For azide 2b, the NCI plot shows a green isosurface between the hydrogen of the OH group and N^3^, indicating the presence of a weak interaction. Within the NCI framework, the OH⋯N interaction in 2-aminoethanol, with no bcp, was characterized as a weak hydrogen bond by the presence of a blue-green isosurface between the OH and NH_2_ moieties, which is different from that observed for 2b.^46,47^ Also, the NCI plot shows a similar green isosurface between the oxygen of the OH group and the hydrogen atom H^2^ in the 2a regioisomer (*d*_H⋯O_ = 2.37 Å; C^2^H^2^⋯O = 97°), which indicates it not being a hydrogen bond.^48^

In 2b, the charge of N^3^ (−0.415 *e*) becomes more negative than in 1b, due to the interaction with the hydrogen atom (+0.594 *e*). Also, in 2a the charge of H^2^ is more positive than in 1a due to the contact with the oxygen of the OH group. Thus, it would be more appropriate to characterize the contact between OH and N^3^ in 2b as an attractive electrostatic interaction instead of a hydrogen bond.

In the regioisomer 3a, one of the methyl group of the TMS moiety is oriented forward of the azide group (*d*_H⋯N^3^_ = 2.90 Å; CH⋯N^3^ = 121°), and a bcp is found between N^3^ and a hydrogen atom of the TMS. Also, the NCI plot shows a green isosurface between these atoms, denoting a weak interaction. In the regioisomer 3b, one of the hydrogen atoms of the methyl group of TMS is directed towards N^3^ (*d*_H⋯N^3^_ = 3.00 Å; CH⋯N^3^ = 100°), but no bcp was found for this interaction, though a green NCI isosurface was visualized. In both 3a and 3b regioisomers, there was an attractive interaction between the oxygen atom of the OTMS group and H^2^ (*d*_H_2_⋯O_ = 2.43 Å for 3a; *d*_H_2_⋯O_ = 2.54 for 3b), reflecting the presence of a green NCI isosurface similar to those found in azide 2, which might determine the orientation of the C–O bond relative to the double bond. Also, the charges of the H^2^ atom in 3a and 3b were more positive than in 1a and 1b, respectively, due to the interaction with the oxygen atom of the OTMS group. These weak attractive interactions affect the structure of the regioisomers, whereby in 3a the bulky TMS group is pointing inside the double bond, while in 3b it is far off the double bond, making this structure less sterically congested. Therefore, it could be assumed that the structure of 3a is disfavoured by steric repulsion, which influences the equilibrium distribution.

These interactions that are visualized as a green NCI isosurface between a hydrogen of the phenyl ring and H^2^ (*d*_H_2_⋯N_ = 2.20 Å) for 4a and between a hydrogen of the aromatic ring and N^3^ in 4b were repulsive in the first case and attractive in the second. The influence of this kind of interaction was negligible with respect to the conjugative effect, as was established and we will analyse in detail below.

Several stereoelectronic effects contribute to the subtle energy differences. It was stated that populations of regioisomers during the interconversion by the allylic azide rearrangement are governed by stabilization of the C=C bonds with more substitutions.^31^ The nature of the stabilizing interactions, such as donor–acceptor interactions, could be easily rationalized through the NBO analysis.

The hyperconjugative interactions in which π*C=C and πC=C of the double bond of the allylic group act as acceptor and donor orbitals, respectively, were examined according to the second-order perturbation energy (*E*^(2)^) in the NBO analysis. Also, other selected interactions were analysed. The second order stabilization energies *E*^(2)^ associated with the most relevant hyperconjugative interactions in the 1–4 azides are summarized in Table 2.

Second-order perturbation energies (*E*^(2)^, kcal mol^−1^) of the main donor–acceptor interactions in the 1–4 azides^a^

**Table:** 

Donor	Acceptor	1a	2a	3a	4a
σC^1^–N^1^	π*C^2^=C^3^	2.91	3.00	3.27	3.13
σC^1^–H^1^		4.97	4.98	4.78	4.95
σC^4^–H^4^		13.27	13.52	13.40	—
σC^4^–C^5^		—	—	—	17.52
ηN^1^		1.24	1.29	1.56	1.43
σZa→π*C ^**2**^ **=C** ^**3**^		**22.39**	**22.79**	**23.01**	**27.03**
πC^2^=C^3^	σ*C^1^–N^1^	6.65	6.66	6.82	6.58
	σ*C^1^–H^1^	2.84	2.71	2.56	2.68
	σ*C^4^–H^4^	6.30	6.03	5.96	—
	σ*C^4^–C^5^	—	—	—	13.83
**πC** ^**2**^ **=C** ^**3**^ →σ*Z_**b**_		**15.79**	**15.40**	**15.34**	**23.09**

a σZ_a_ and σ*Z_b_ denote the bonding and antibonding orbitals that interact with the π*C=C and πC=C orbitals of the allylic group, respectively.

**Table:** 

Donor	Acceptor	1b	2b	3b	4b
σC^3^–N^3^	π*C^1^=C^2^	3.12	3.00	2.92	2.89
σC^3^–C^4^		3.42	3.45	3.22	3.17
ηN^1^		1.65	1.50	1.32	1.64
σC^4^–C^5^		—	—	—	0.59
**σZ**_**a**_ →π*C ^**2**^ **=C** ^**3**^		**8.19**	**7.95**	**7.46**	**8.29**
πC^1^=C^2^	σ*C^3^–N^3^	6.48	6.52	7.08	6.22
	σ*C^3^–C^4^	3.18	3.31	3.09	3.10
**πC** ^**1**^ **=C** ^**2**^ →σ*Z_**b**_		**9.66**	**9.83**	**10.17**	**9.32**
ηN^3^	σ*O–H	—	0.68	—	—

In the primary azides 1–3a there were hyperconjugative interactions among σC^1^–N^1^, σC^1^–H^1^, σC^4^–H donor orbitals and π*C^2^=C^3^ antibonding orbital as the acceptor. Also, there was a hyperconjugative interaction ηN^1^→π*C^2^=C^3^ with lower values of *E*^(2)^. The stronger hyperconjugative effect was associated with the interactions between the two out-of-plane σC^4^–H^4^ sigma bonds and the π*C^2^=C^3^ antibonding orbital. For 1a and 2a, the *E*^(2)^ of the σZ_a_→π*C^2^=C^3^ interactions were similar, while it is a little higher for 3a. For 1a, when the πC^2^=C^3^ orbitals acted as donors, the energy of πC^2^=C^3^→Z_b_ was a little stronger than that for 2a and 3a.

The energy of the σZ_a_→π*C^2^=C^3^ interactions showed a slightly higher value for 1b and did not justify the shift of the population equilibrium. In 2b, the *E*^(2)^ of πC^2^=C^3^→σ*C^3^–N^3^ and πC^2^=C^3^→σ*C^3^–C^4^ were slightly more favourable than in 1b. Also, 2b showed a ηN^1^→σ*O–H (0.68 kcal mol^−1^) interaction, although this only weakly provided stabilization to this regioisomer, as was reported for other systems.^49^

In the secondary azide 3b, the energy of πC^1^=C^2^→σ*C^3^–N^3^ was higher than in 1b by 0.6 kcal mol^−1^, but the energies of πC^1^=C^2^→σ*C^3^–C^4^ were quite similar for both. Therefore, the shift in azide 3's equilibrium towards the secondary azide, compared with azide 1, might be explained by the more favourable hyperconjugative πC^1^=C^2^→σ*C^3^–N^3^ interaction. It was also noticeable that in 3b there was less steric repulsion than in 3a due to the orientation of the bulky substituent group.

We might assume that the interactions between the N^3^ atom and oxygen of the OH and the OTMS groups in 2b and 3b, respectively, govern the structure of the molecule, giving a more favourable orbital alignment for the πC^1^=C^2^→σ*C^3^–N^3^ interaction, *i.e.* both types of interactions act cooperatively to provide more stabilization to these regioisomers.

In the regioisomer 4a, there were stronger conjugative interactions πC^4^–C^5^→π*C^2^–C^3^ (17.52 kcal mol^−1^) and πC^2^–C^3^→π*C^4^–C^5^ (13.83 kcal mol^−1^), while in 4b, these π-conjugation between their vicinal multiple bonds were absent. Thus, the conjugative effect was responsible for the displacement towards the regioisomer 4a, which was confirmed by the NBO analysis.

### Primary *vs.* tertiary azides

The computed relative energies (Δ*G*) and the equilibrium ratio for the regioisomers 5–7 are listed in Table 3. In azides 5, 6 and 7, there was an additional methyl group compared with the azides 1, 2 and 4, respectively, and these showed a slight shift towards the primary azides.

**Table:** Relative free energies (Δ*G*, kcal mol^−1^) and calculated and experimental equilibrium ratios for the azides under study^a^

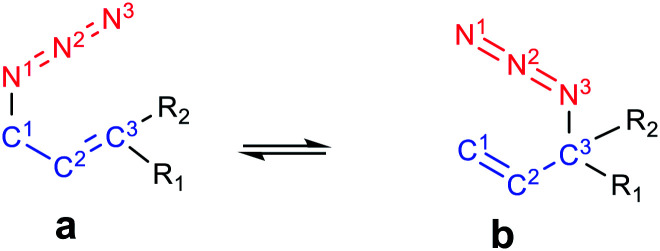					
Azide (*X*)	R_1_	R_2_	Δ*G* (*X*_b_ − *X*_a_)	*X*_a_ : *X*_b_ ratio	
				Calcd^a^	Exp.^b^
5	Me	Me	1.45	92 : 8	87 : 2330
6	CH_2_OH	Me	−0.06	48 : 52	55 : 4522
7	Ph	Me	3.65	100 : 0	100 : 030

a Ratios were computed using Boltzmann factors based on Δ*G*.

b 6a (47% *E-trans*, 8% *Z-cis*).

The primary azides 5a and 7a were 1.45 and 3.65 kcal mol^−1^ more stable than the corresponding tertiary isomers, respectively, being the estimated equilibrium ratios concordant with the experimental results. For azide 6, substituted with a hydroxyl group, the difference in energy between the primary and tertiary regioisomer was very small and the calculated ratio slightly favoured the latter in contrast with the experimental result. However, this result reflects an equilibrium shift in azide 6 with respect to azide 5, which is in agreement with the calculated values previously reported.^23^

**Fig. 3:**
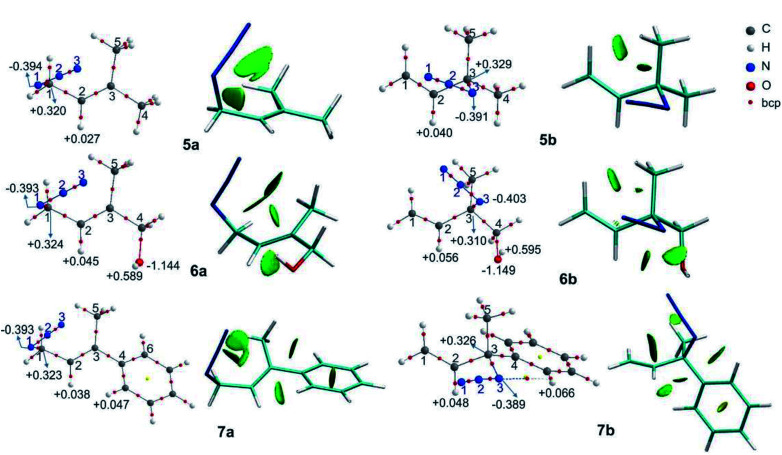
Molecular graphs of the azides 5–7 (left). For selected atoms, the atomic charges *q* (Ω) are given in *e*. NCI gradient isosurfaces (right), represented at an isovalue of 0.5 a.u. and blue-green-red colour scale from −0.05 < sign(*λ*_2_)*ρ* < +0.05 a.u.

The molecular graphs of azides 5–7 are depicted in Fig. 3 (the topological properties evaluated at the bcps are listed in Table S4 in the ESI†) along with their NCI plots.

All the regioisomers showed a close contact between H^1^ and H^5^ (*d*_H^1^⋯H^5^_, 5a = 2.07 Å, 5b = 2.33 Å, 6a = 2.07 Å, 6b = 2.27 Å, 7a = 2.08 Å, 7b = 2.32 Å), and the NCI plots displayed a green isosurface between them, indicating that it was a weak repulsive interaction. These isosurfaces were larger in the primary azides, in agreement with the distances H^1^–H^5^.

In regioisomers 6a and 6b, there was a weak interaction between the oxygen atom of the OH group and H^2^ (*d* = 2.39 and 2.53 Å, respectively), as evidenced by the green isosurface in the NCI plot. Also, the charges of H^2^ in 6a and 6b were more positive than in 5a and 5b, as a consequence of the interaction with the oxygen atom.

In azide 6b, the OH group is directed towards N^3^ (*d*_H⋯N_ = 2.38 Å; OH–N^1^ = 107°), and no bcp was observed between the hydrogen atom of the OH group and the N^3^, but a green isosurface was found in the NCI plot, indicating a weak interaction. Also, the charges of N^3^ (−0.403 *e*) and of the hydrogen atom of the OH group (+0.595 *e*) were more negative and positive than those in 5b and 6a, respectively.

In azide 7a, there were repulsive interactions between two hydrogen atoms of the aromatic ring and the H^2^ and a hydrogen of the methyl group (C^5^H_3_). While in azide 7b, an attractive interaction between N^3^ and a hydrogen of the phenyl group was observed. For this interaction, a bcp was found and the topological parameters reflected a weak interaction, in agreement with the green NCI isosurface between both atoms. Thus, the primary azide 7a was disfavoured due to the steric congestion; however, it predominated in the equilibrium as a consequence of the conjugation effect (see below).

The second-order perturbation energy (*E*^(2)^) values of the relevant hyperconjugative interactions are summarized in Table 4.

Second-order perturbation energies (*E*^(2)^, kcal mol^−1^) of the main donor–acceptor interactions in the 5–7 azides^a^

**Table:** 

Donor	Acceptor	5a	6a	7a
σC^1^–N^1^	π*C^2^=C^3^	2.36	2.42	2.67
σC^1^–H^1^		5.11	5.14	4.64
σC^4^–H^4^		14.07	14.12	—
σC^4^–C^6^				13.17
σC^5^–C^7^				0.61
σC^5^–H^5^		15.26	15.27	14.30
ηN^1^		0.97	1.03	1.15
**σZ**_**a**_ →π*C ^**2**^ **=C** ^**3**^		**37.77**	**37.98**	**36.54**
πC^2^=C^3^	σ*C^1^–N^1^	7.48	7.25	7.22
	σ*C^1^–H^1^	2.10	2.06	1.96
	σ*C^4^–H^4^	6.61	5.93	
	σ*C^4^–C^6^			12.12
	σ*C^5^–C^7^			1.02
	σ*C^5^–H^5^	5.91	5.90	5.67
**πC** ^**2**^ **=C** ^**3**^ **→σZ**_**b**_		**22.10**	**21.14**	**27.99**

a σZ_a_ and σ*Z_b_ denote the bonding and antibonding orbitals that interact with the π*C=C and πC=C orbitals of the allylic group, respectively.

**Table:** 

Donor	Acceptor	5b	6b	7b
σC^3^–N^3^	π*C^1^=C^2^	3.23	3.19	3.31
σC^3^–C^4^		3.76	3.60	3.25
ηN^1^		1.69	1.61	1.73
**σZ**_**a**_ →π*C ^**2**^ **=C** ^**3**^		**8.68**	**8.40**	**8.28**
πC^1^=C^2^	σ*C^3^–N^3^	6.01	7.01	5.82
	σ*C^3^–C^4^	3.28	2.96	3.40
**πC** ^**1**^ **=C** ^**2**^ **→σZ**_**b**_		**9.29**	**9.97**	**9.22**
ηN^3^	σ*O–H		0.65	

In the primary azides 5a and 6a, there were stronger hyperconjugative interactions between the two out-of-plane σC^4^–H^4^ and σC^5^–H^5^ orbitals and the π*C^2^=C^3^ antibonding orbital, and the energies of interaction σZ_a_→π*C^2^=C^3^ for 5a (37.77 kcal mol^−1^) and 6a (37.98 kcal mol^−1^) were substantially similar.

The interactions wherein the πC^2^=C^3^ acts as the donor orbital were a little stronger in 5a than in 6a, particularly the πC^2^=C^3^→σC^4^–H^4^ interactions. In 5a, the σ*C^4^–H^4^ orbitals were better aligned to interact with the πC^2^=C^3^ donor orbital, while in 6a the contact between the oxygen atom of the OH group and H^2^ affected the orientation of these orbitals, and thus the overlap among them. Thus, the values of *E*^(2)^ for the πC^2^=C^3^→σZ_b_ interactions were slightly higher in azide 5a.

Comparing the energies of σZ_a_→π*C^2^=C^3^ and πC^2^=C^3^→Z_b_ between azides 5a, 6a and 1a, 2a, those of 5a, 6a considerably higher, which clearly demonstrated the relevance of the hyperconjugation, as reflected in the equilibrium ratio.

In the tertiary azides 5b and 6b the energies of σZ_a_→π*C^2^=C^3^ were almost similar. When the πC^1^=C^2^ orbital acted as a donor, the energies of πC^1^=C^2^→σ*Z_b_ were higher in 6b, particularly the πC^1^=C^2^→σ*C^3^–N^3^ interaction, which was stronger by 1 kcal mol^−1^ than in 5b. Also, in 6b, the interaction ηN^3^→σ*O–H (0.65 kcal mol^−1^) was found, as in azide 2b. The stabilizing ηN^3^→σ*O–H interaction led to a better πC^1^=C^2^→σ*C^3^–N^3^ orbital orientation for their overlap, and thus both interactions contributed to stabilizing the regioisomer 6b.

In 7a, the phenyl group is twisted out of the plane to relieve the repulsive interaction with the methyl (C^5^H_3_) group (C^2^–C^3^–C^4^–C^6^ = 37°) and this disrupted the conjugation. A stronger πC^4^=C^6^→π*C^2^=C^3^ interaction (13.17 kcal mol^−1^) and another weak πC^5^–C^7^→π*C^2^=C^3^ (0.61 kcal mol^−1^) were found, but the energy of σZ_a_→π*C^2^=C^3^ was lower than those for azides 5a and 6a. When πC^2^=C^3^ acted as an orbital donor, a stronger conjugation was found for πC^2^=C^3^→π*C^4^=C^6^ (12.12 kcal mol^−1^), with the energies of πC^2^–C^3^→σ*Z_b_ (27.99 kcal mol^−1^) being higher than in 5a and 6a. Also, in 7b, the energies of σZ_a_→π*C^2^=C^3^ and πC^1^=C^2^→Z_b_ were weaker than in 5b and 6b.

Although the conjugation energy of 7a was weakened by geometric constraints and it was decreased compared to that in 4b, it was still strong enough to explain the observed equilibrium shift.

### Secondary *vs.* tertiary azides

**Fig. 4:**
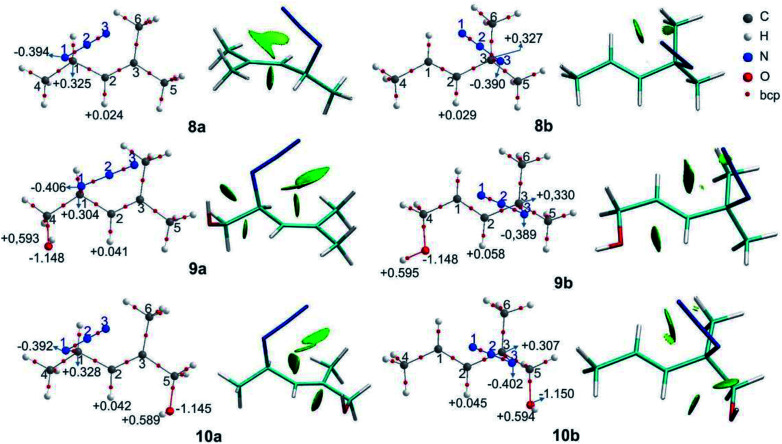
Molecular graphs of the azides 8–10 (left). For selected atoms, the atomic charges *q* (Ω) are given in *e*. NCI gradient isosurfaces (right), represented at an isovalue of 0.5 a.u. and blue-green-red colour scale from −0.05 < sign(*λ*_2_)*ρ* < +0.05 a.u.

The relative free energies (Δ*G*) and the equilibrium ratios for the regioisomers 8–12 are listed in Table 5. The molecular graphs of the azides 8–10 are depicted in Fig. 4 along with their NCI plots. (For the remaining azides, see the ESI†).

**Table:** Relative free energies (Δ*G*, kcal mol^−1^) and calculated and experimental equilibrium ratios for the azides under study^a^

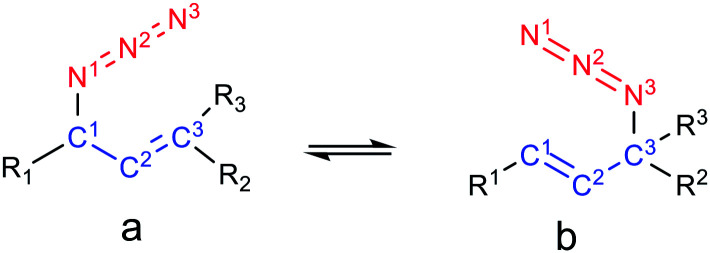						
Azide (*X*)	R_1_	R_2_	R_3_	Δ*G* (*X*_b_ − *X*_a_)	*X*_a_ : *X*_b_ ratio	
					Calcd^a^	Exp.^b^^,^^c^
8	Me	Me	Me	0.57	72 : 28	65 : 35 (ref. 22)
9	CH_2_OH	Me	Me	1.93	96 : 4	82 : 18 (ref. 22)
10	Me	CH_2_OH	Me	−0.31	37 : 63	36 : 64 (ref. 50)
11	*cHex*	CH_2_OH	Me	−1.03	15 : 85	37 : 63 (ref. 50)
12	Ph	CH_2_OH	Me	−3.6	0 : 100	0 : 100 (ref. 50)

a Ratios were computed using Boltzmann factors based on Δ*G*.

b 8b (32% *trans*, 3% *cis*).

c The experimental data given correspond to a structurally similar allylic azide to 12, wherein the aromatic group is 2-pyridyl instead phenyl.

The secondary azides 8a and 9a were more stable than their corresponding tertiary regioisomers by 0.6 and 1.9 kcal mol^−1^, respectively; therefore, the more substituted alkene isomers were thermodynamically favoured, as stated previously.^31^ This is the reverse for azides 10, 11 and 12, being secondary regioisomers less stable by 0.3, 1.0 and 3.6 kcal mol^−1^ than their tertiary counterparts, respectively. Overall, the calculated equilibrium ratios acceptably reproduced the experimental results and reflected the observed regioisomer population composition changes with respect to azide 8.

In azide 9a, the OH group is directed towards N^1^ (*d*_H⋯N_ = 2.38 Å; OH–N^1^ = 106°), and in azides 10–12b it points towards N^3^ (*d*_H⋯N_ = 2.33 Å; OH–N^3^ = 106°), forming a stabilizing weak interaction, as denoted by a green NCI isosurface. Again, from the molecular graphs, no bcp was observed between the hydrogen atom of the OH group and N^1^ in azide 9a and N^3^ in azides 10–12b.

In 9a and 9b, the negative/positive charges of the O/H atoms of the OH group were similar; nevertheless, the negative charge of N^1^ in 9a was higher than that in the other secondary azides. In 10b, the positive/negative charges of the H/O atoms of the OH group were augmented with respect to those in 10a. Also, the negative charge of N^3^ in 10b was higher than that in 8b and 9b. Similar results were found for azides 11b and 12b. Thus, the polarization of the O–H bond and the increase in the electronic population in N^3^ arise from the contact between these atoms. In all the regioisomers, a contact between the oxygen atom and H^2^ was visualized by a green NCI isosurface.

The second-order perturbation energy of the relevant hyperconjugative interactions are summarized in Table 6.

Second-order perturbation energies (*E*^(2)^, kcal mol^−1^) of the main donor–acceptor interactions in the 8–12 azides

**Table:** 

Donor	Acceptor	8a	9a	10a	11a	12a
σC^1^–N^1^	π*C^2^=C^3^	2.36	2.17	2.47	2.56	2.31
σC^1^–C^4^		3.25	3.14	3.26	2.74	2.88
σC^1^–H^1^			0.74			
σC^5^–H^5^		14.08	14.20	14.12	14.22	14.12
σC^6^–H^6^		15.34	15.32	15.30	15.33	15.30
ηN^1^		1.15	0.81	1.22	1.32	1.26
σC^3^–C^4^					0.61	
**σZ**_**a**_ →π*C ^**2**^ **=C** ^**3**^		**36.18**	**36.38**	**36.37**	**36.78**	**35.87**
πC^2^=C^3^	σ*C^1^–N^1^	7.74	8.42	7.45	7.56	7.21
	σ*C^1^–C^4^	2.20	1.81	2.31	2.27	2.47
	σ*C^1^–H^1^	0.58	0.98			
	σ*C^5^–H^5^	6.65	6.58	5.96	5.99	5.88
	σ*C^6^–H^6^	5.92	5.93	5.82	5.79	5.78
**πC** ^**2**^ **=C** ^**3**^ →σ*Z_**b**_		**23.09**	**23.72**	**21.54**	**21.61**	**21.34**

**Table:** 

Donor	Acceptor	8b	9b	10b	11b	12b
σC^3^–N^3^	π*C^1^=C^2^	3.07	3.16	3.02	3.16	3.06
σC^3^–C^6^		3.66	3.67	3.50	3.55	3.87
σC^4^–H^4^		13.26	13.61	13.32		
ηN^1^		1.53	1.56	1.43	1.42	1.44
σC^4^–C					8.83	17.65
**σZ**_**a**_ →π*C ^**2**^ **=C** ^**3**^		**21.52**	**22.00**	**21.27**	**16.96**	**26.02**
πC^1^=C^2^	σ*C^3^–N^3^	6.56	6.49	7.69	8.01	7.09
	σ*C^3^–C^5^	3.40	3.32	3.03	2.90	3.17
	σ*C^4^–H^4^	6.37	6.09	6.32		
	σ*C^4^–C				6.43	14.16
**πC** ^**1**^ **=C** ^**2**^ →σ*Z_**b**_		**16.33**	**15.90**	**17.04**	**17.34**	**24.42**
ηN^3^	σ*O–H			0.69	0.66	0.66

From the NBO analysis, it was observed that in the secondary azides 8–12a, there were hyperconjugative interactions among the donor σC^1^–N^1^, σC^1^–C^4^, σC^5^–H^5^ and σC^6^–H^6^ and the π*C^2^=C^3^ antibonding orbital as the acceptor. In 9a, a weak σC^1^–H^1^→π*C^2^=C^3^ interaction (0.74 kcal mol^−1^) and in 11a a σC^4^C→π*C^2^=C^3^ interaction (0.61 kcal mol^−1^) were also found due to their orientation. The energies of the σZ_a_→π*C^2^=C^3^ interactions were similar for the secondary azides and do not explain the observed changes in the equilibrium populations.

There are some significant differences in energies when the orbital πC^2^=C^3^ acts as a donor. Overall, the πC^2^=C^3^→σ*Z_b_ interactions were weaker in azides 10–12a than in 8a (by about 1.5 kcal mol^−1^), particularly the πC^2^=C^3^→σ*C^5^–H^5^ interactions. In azides 10–12a the OH group is attached to C^5^ and this might affect the πC^2^=C^3^→σ*C^5^–H^5^ interaction. The πC^2^=C^3^→σ*C^1^–H^1^ interaction was only found in azides 10a and 11a. For azide 9a, πC^2^=C^3^→σ*N^1^–C^1^ was slightly stronger, which might be a consequence of the contact between the hydrogen atom of the OH group and the N^1^ atom, even though for this case no hyperconjugation interaction was found. Thus, the strength of the hyperconjugative interactions in which the πC^2^=C^3^ act as donor orbital decrease when the CH_2_OH group is attached to one carbon atom of the double bond.

In the tertiary azides 8b–12b, there were hyperconjugative interactions among the σC^3^–N^3^, σC^3^–C^5^ orbital donor and π*C^1^=C^2^ orbital acceptor. Also, azides 8b–10b displayed σC^4^–H^4^→π*C^1^=C^2^ interactions, while 11b and 12b showed σC^4^–C→π*C^1^=C^2^ interactions involving the cyclic substituent. The σC^4^–C→π*C^1^=C^2^ interaction in azide 11b had a lower energy than similar interactions in the other azides, but this did not explain the predominance of the regioisomer 11b in the equilibrium mixture. Also, the energies of σZ_a_→π*C^2^=C^3^ did not reflect the trend of the tertiary azides population in the equilibrium mixture.

The hyperconjugative energies in 9b, wherein the πC^1^=C^2^ acts as a donor orbital, were slightly weaker than in 8b, due to the CH_2_OH being attached to the double bond, as was mentioned above.

The energies of πC^1^=C^2^→σ*Z_b_ were higher in 10b and 11b than in 8a, mainly because of the strengthening of the πC^1^=C^2^→σ*C^3^–N^3^ interaction (by 1.13 and 1.45 kcal mol^−1^, respectively). In both 10b and 11b, ηN^3^→σ*O–H interactions (0.69 and 0.66 kcal mol^−1^, respectively) were also found. This interaction, besides being a stabilizing one, also affects the orientation of the regioisomers, favouring the overlap of the πC^1^=C^2^ and σC^3^–N^3^ orbitals.

**Fig. 5:**
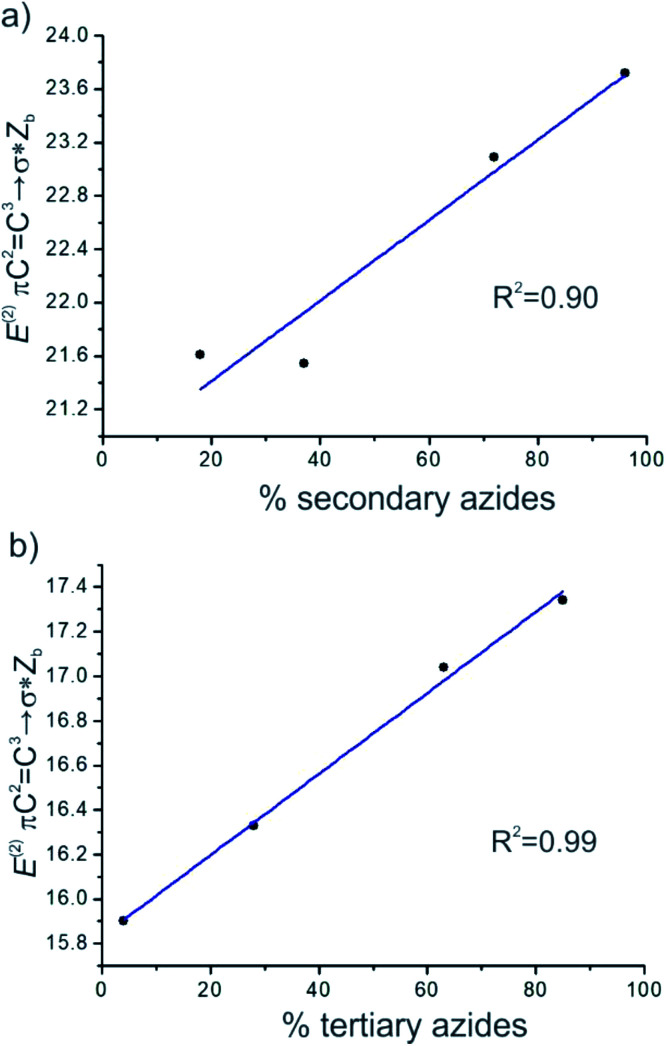
Plots of the correlation between (a) the hyperconjugation energies *E*^(2)^ πC^2^=C^3^→σ*Z_b_ and the percentage of secondary azides and (b) *E*^(2)^ πC^1^=C^2^→σ*Z_b_ and the percentage of tertiary azides in the equilibrium mixture.

It was noted that the greater the energies of πC^2^=C^3^→σ*Z_b_ and πC^1^=C^2^→σ*Z_b_, the more the equilibrium was shifted to the secondary and tertiary azides isomers, respectively. A good linear correlation between *E*^(2)^ πC^2^=C^3^→σ*Z_b_ and the calculated percentage of secondary azides was obtained for azides 8–11 (*R*^2^ = 0.90). Also, a better linear correlation between *E*^(2)^ πC^1^=C^2^→σ*Z_b_ and the percentage of tertiary azides was found (*R*^2^ = 0.99) (Fig. 5).

According to these results, the hyperconjugative interactions in which the double bond acts as a donor were the main factors that controlled the stabilization of the regioisomers, and therefore the distribution of the regioisomers in the equilibrium mixture, although other electrostatic interactions may also influence the process.

In azide 12a, the energies of the πC^1^=C^2^→σ*C^3^–N^3^ and πC^2^=C^3^→σ*Z_b_ interactions were the lowest. The conjugated azide 12b was greatly stabilized by the conjugative interactions C^4^–C→π*C^1^=C^2^ (17.65 kcal mol^−1^) and πC^1^=C^2^→σC^4^–C (14.16 kcal mol^−1^). Also, there was an interaction ηN^3^→σ*O–H, which favoured the πC^1^=C^2^→σC^3^–N^3^ interaction. Both interactions contributed to stabilizing the structure, but the governing factor in this case was the conjugative effect.

## Conclusions

In this work, several representative allylic azides with different degrees of substitution on the double bond were studied using density functional theory and QTAIM, NCI, and NBO approaches in order to evaluate the factors responsible for their stabilities and therefore to explain the observed equilibrium shift.

**Fig. 6:**
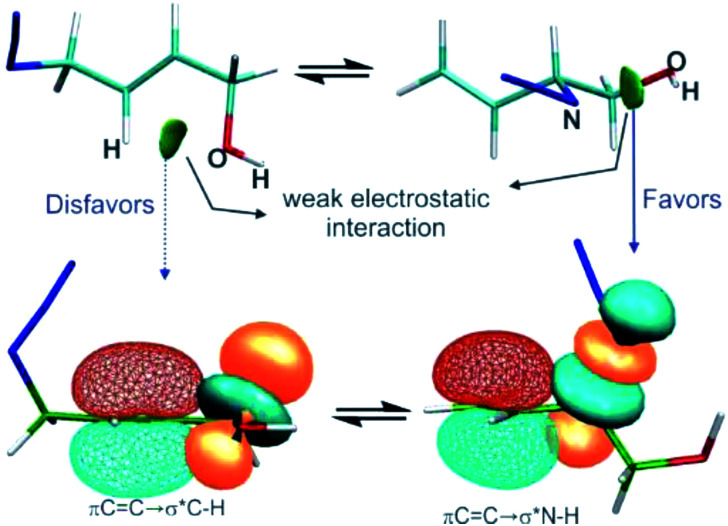
The interplay of the electrostatic interactions (top, visualised through NCI gradient isosurfaces at an isovalue = 0.5) and hyperconjugative interactions (bottom, displayed by natural bond orbitals at an isovalue = 0.05) in azide regioisomers substituted with a hydroxyl group.

The results revealed that when the azides were substituted with OH groups or heteroatoms, weak electrostatic interactions could be evidenced in each regioisomer that influence the conformation and thus the hyperconjugative interactions (Fig. 6).

In the regioisomer in which the substituent group was attached to a carbon atom of the double bond, an interaction was formed between the O atom of the OH group and an H atom of the allylic moiety and this weakened the hyperconjugation πC=C→σ*Z_b_. In the other regioisomer, the OH group was close to the azide group, and an interaction was observe between them which gave a more favourable orbital alignment for the negative hyperconjugative interaction πC=C→σ*Z_b_. The analysis of the charge density distribution showed that the interaction between the OH group and a nitrogen atom of the azide group showed the characteristic of an electrostatic attractive interaction, rather than a hydrogen bond as was previously proposed and this was attributed to be responsible for the observed equilibrium shift.

The equilibrium shift could not be explained by a specific interaction in one of the regioisomers, instead it was due to a combination of two opposite effects: weakening and strengthening of the hyperconjugation promoted by the electrostatic interactions involving the substituent group.

Therefore, hyperconjugative interactions were found to play a primary role in the regioisomers stability. A good linear correlation was obtained for the secondary and tertiary azides in equilibrium between the hyperconjugative energies of πC=C→σ*Z_b_ and the calculated percentage of secondary and tertiary azides. Also, it was observed that other effects, such as steric effects, influenced the stability of the regioisomers.

For the azides substituted with an aromatic ring, the energy of the conjugative interactions provided a great stabilization to the conjugated regioisomer and this was enough to explain its exclusive existence in the equilibrium mixture. This effect dominated over other kinds of interactions, such as electrostatic interactions or steric factors.

This study not only provides insights into the factor controlling the stabilities of substituted allylic azides, but also allows one to predict which regioisomer will be predominant in the equilibrium mixture.

## Conflicts of interest

There are no conflicts to declare.

## Supplementary Material

RA-010-C9RA10093H-s001
