# Supplementary material for: Insight into the factors controlling the equilibrium of allylic azides
Source: RSC Adv. 2020 Jan 27;10(8):4404–13. doi: 10.1039/c9ra10093h (PMC9049130; doi:10.1039/c9ra10093h)
Supplement: RA-010-C9RA10093H-s001 [file RA-010-C9RA10093H-s001.pdf]

Electronic Supplementary Material (ESI) for RSC Advances.
This journal is © The Royal Society of Chemistry 2019

SUPPORTING INFORMATION

Insight on the factors controlling the equilibrium of allylic azides

Margarita M. Vallejos,[a,*] and Guillermo R. Labadie[b,c]

[a]Laboratorio de Química Orgánica, IQUIBA-NEA, Universidad Nacional del Nordeste, CONICET, FACENA, Av. Libertad 5460, Corrientes 3400, Argentina E-mail: vallejos.marga@gmail.com; m.vallejos@conicet.gov.ar; Tel: +54-379-4457996 ext. 104.

[b]Instituto de Química Rosario, UNR, CONICET, Suipacha 531, S2002LRK, Rosario, Argentina.

[c]Departamento de Química Orgánica, Facultad de Ciencias Bioquímicas y Farmacéuticas, Universidad Nacional de Rosario, Suipacha 531, S2002LRK, Rosario, Argentina

List of contents

- Computational methods. Relative Gibbs free energies computed using different levels of theories for the regioisomers **1** and **2** and calculated equilibrium ratio. (**Table S1**). Page S2.
- Free energy profiles for the [3,3]-sigmatropic rearrangement of allylic azides **1-4**. (**Figure S1**). Page S3.
- Free energy profiles for the [3,3]-sigmatropic rearrangement of allylic azides **5-7**. (**Figure S2**). Page S4.
- Free energy profiles for the [3,3]-sigmatropic rearrangement of allylic azides **8-12**. (**Figure S3**). Page 5.
- Local topological properties for the selected bond critical point and delocalization indexes (*DI*) for azides **1-4** (**Table S2**). Pages S6 and S7
- Net charges for selected atoms (in e) in azides **1-4**. (**Table S3**). Page S7.
- Local topological properties for azides **5-7** (**Table S4**). Page S8.
- Net charges for selected atoms in azides **5-7**. (**Table S5**). Page S8.
- Molecular graphs and NCI gradient isosurfaces of the azides **11** and **12**. (**Figure S4**). Page S9.
- Local topological properties for the selected bond critical point and delocalization indexes (*DI*) for azides **8-12** (**Table S6**). Pages S9 and S10.
- Net charges for selected atoms (in e) in azides **8-12**. (**Table S7**). Page S10-
- Cartesian coordinates. Pages S11-S17.
- References. Page S18.

Computational Methods

The geometries of the allylic azides **1** and **2** were optimized using the M06-2X,[1] MPWB1K[2] and B3LYP[3, 4] functionals together with the 6-31+G(d,p) and 6-311++G(d,p) basis set. The frequency calculations were computed at the same level of theory.

Solvent effects in chloroform were taken into account through single-point of the gas phase structures using the SMD-continuum solvation method with the corresponding functionals and 6-311++G(d,p) basis set.[5] In addition, the Grimme's dispersion correction D3,[6] was also considered for the single-point energies calculations. Free energies were computed at 298.15 K and 1 atm. The relative Gibbs free energies and the calculated equilibrium ratio are shown in Table S1.

Table S1.. Relative Gibbs free energies in Chloroform (*Δ*G, in kcal·mol-1) computed using different functional with 6-311++G(d,p) basis set for the regioisomers **1** and **2** and calculated equilibrium ratio.

		M06-2X		M06-2X(D3)		MPWB1K		B3LYP		B3LYP(D3)	
	X	ΔG	Xa:Xb	ΔG	Xa:Xb	ΔG	Xa:Xb	ΔG	Xa:Xb	ΔG	Xa:Xb
A	**1a**	0.00	86:14	0.00	87:13	0.00	94:6	0.00	97:3	0.00	90:10
	**1b**	1.10		1.12		1.68		2.05		1.30	
	**2a**	0.00	49:51	0.00	68:32	0.00	68:32	0.00	84:16	0.00	55:45
	**2b**	-0.02		0.44		0.46		0.99		0.11	
B	**1a**	0.00	83:17	0.00	84:16	0.00	93:7	0.00	98:2	0.00	89:11
	**1b**	0.97		0.98		1.59		2.05		1.27	
	**2a**	0.00	29:71	0.08	47:53	0.00	68:32	0.00	86:14	0.00	59:41
	**2b**	-0.53		0.00		0.45		1.10		0.22	

[1] Using optimized geometries and thermal correction energies obtained with the corresponding functional and A: 6-31+G(d,p) basis set and B: 6-311++G(d,p) basis set . Δ*G* = (*G*Xa-*G*Xb). [c] Ratios were computed using Boltzmann factors based on Δ*G*. [d]Experimental equilibrium ratio 1a:1b = 67:33[7], 2a:2b = 45:55[7].

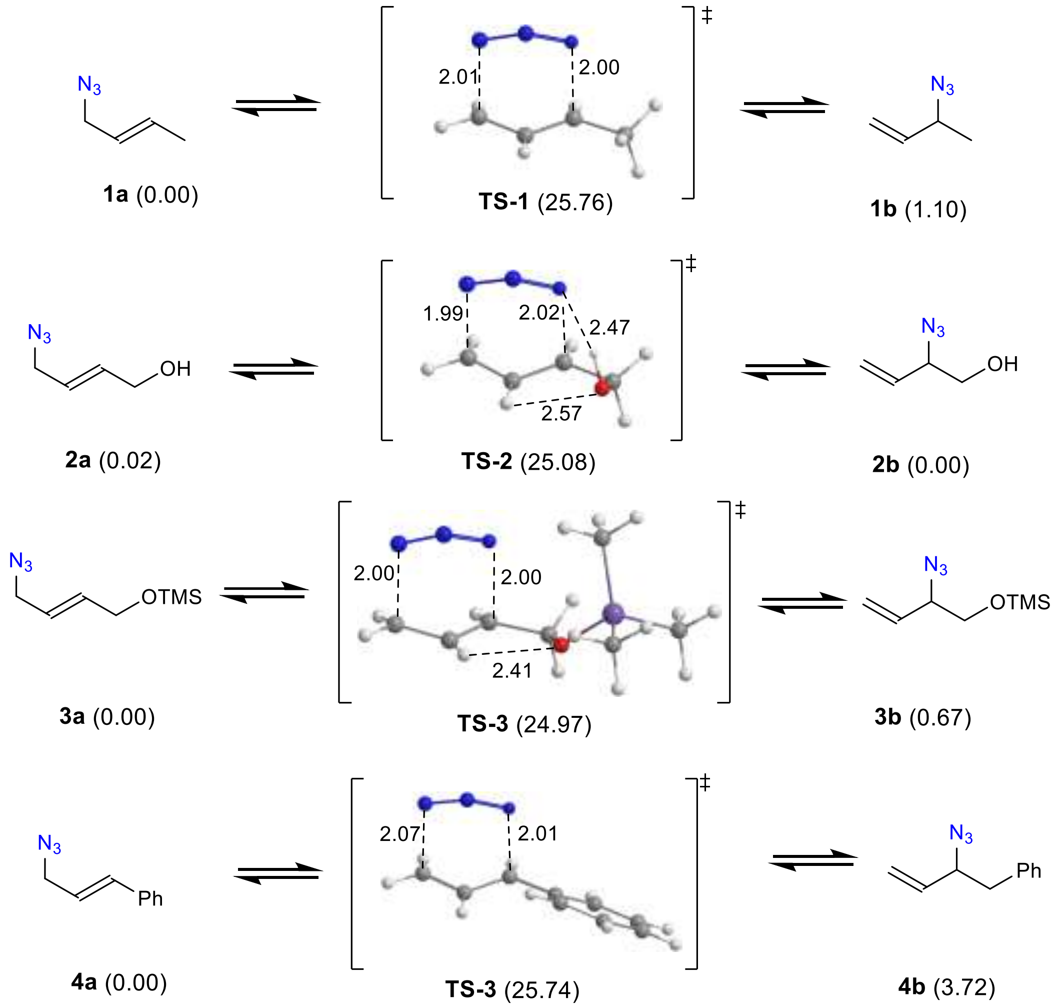

Figure S1. Free energy profiles for the [3,3]-sigmatropic rearrangement of allylic azides **1-4** computed at M06-2X/6-311++G(d,p)SMD//M06-2X/6-31+G(d,p) level. Relative free energies are in kcal/mol. The optimized geometries (in 3D) for the transition structures are showed along with selected distances in Å.

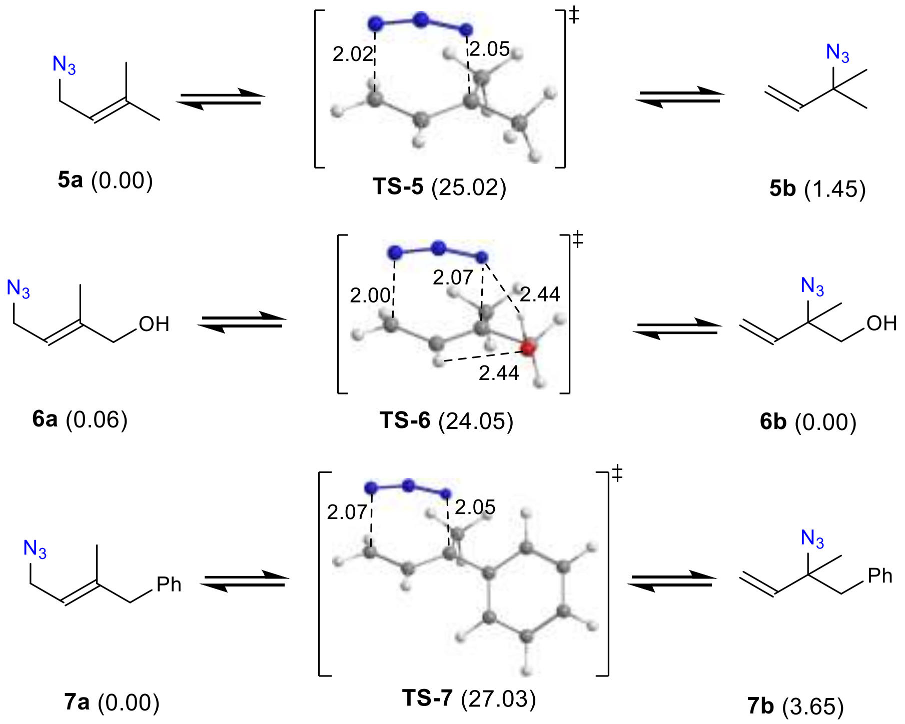

Figure S2. Free energy profiles for the [3,3]-sigmatropic rearrangement of allylic azides 5-**7** computed at M06-2X/6-311++G(d,p)SMD//M06-2X/6-31+G(d,p) level. Relative free energies are in kcal/mol. The optimized geometries (in 3D) for the transition structures are showed along with selected distances in Å.

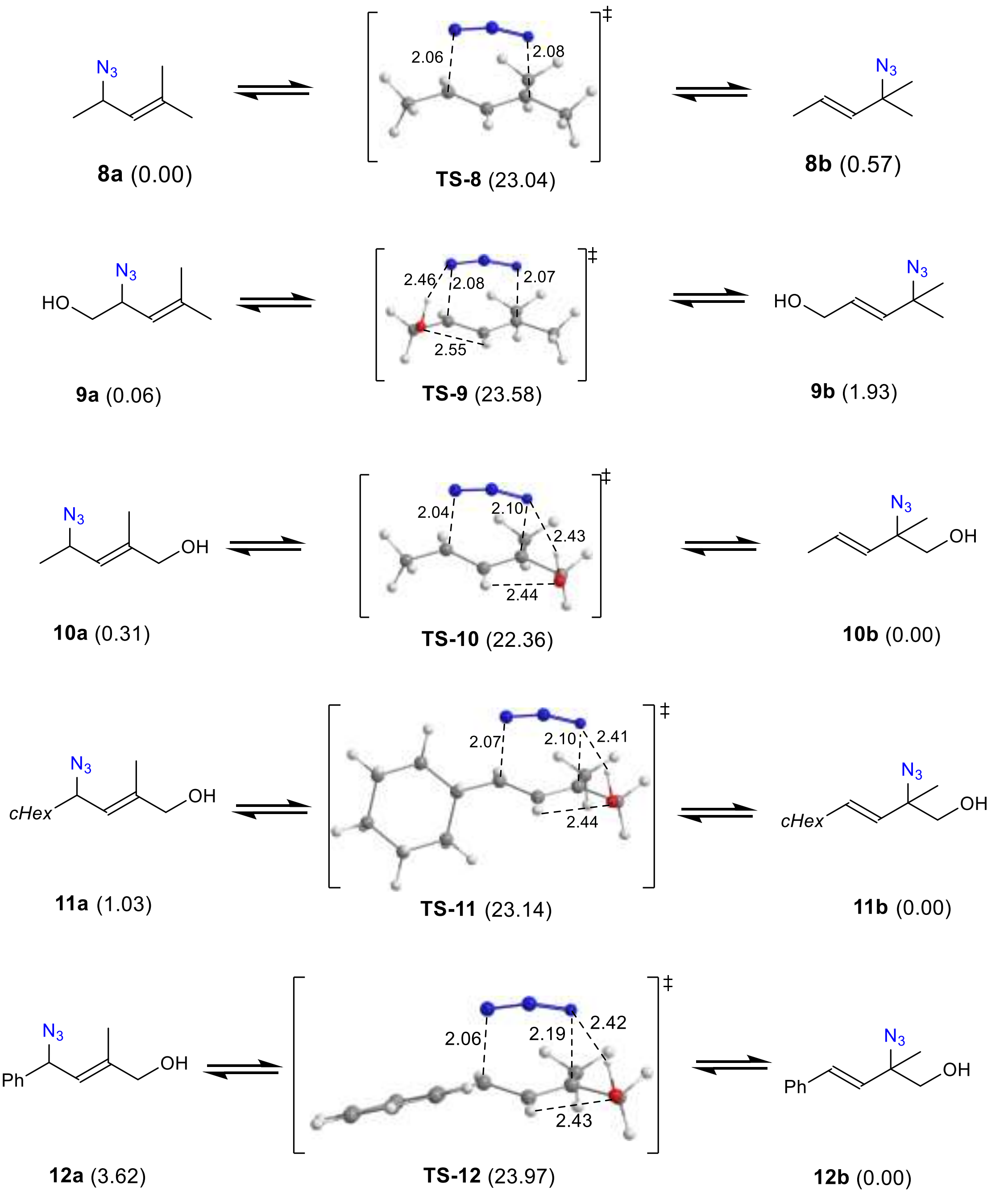

Figure S3. Free energy profiles for the [3,3]-sigmatropic rearrangement of allylic azides **8-12** computed at M06-2X/6-311++G(d,p)SMD//M06-2X/6-31+G(d,p) level. Relative free energies are in kcal/mol. The optimized geometries (in 3D) for the transition structures are showed along with selected distances in Å.

Primary *vs* secondary azides

Topological properties of the charge density – QTAIM

QTAIM analysis is based on the critical points (CP) of the electronic density distribution. Several topological parameters evaluated at the (3,-1) or bond critical point (bcp) provide information about the nature and features of the chemical bonds between the interacting species. The electron density (ρ_b) at the bcp reflects the strength of a bond and its Laplacian ($\nabla^2\rho_b$) measures the local charge concentration ($\nabla^2\rho_b < 0$) or local charge depletion ($\nabla^2\rho_b > 0$).[8, 9] These two properties along with the total energy density (H_b) are used to analyze the covalent character of an interaction[10] In addition, the ellipticity, defined as $\varepsilon = \lambda_1/\lambda_2 - 1$ (where λ_1 and λ_2 are the negative eigenvalues of the Hessian of the electron density with respect to the position, that is, the curvatures of the density at the bcp perpendicular to the bond path), evaluated at bcp gives information about the charge distribution around the bond path and also its stability.[11] Another parameter often used in the study of a bonding interaction is the delocalization index (*DI*). This parameter indicates the extent of exchange of electrons between two atomic basins and it can be calculated between two atoms bonded by a bond path or without having a bond path.

Table S2. Local topological properties for the selected bond critical point and delocalization indexes (*DI*) for azides **1-4**[a]

Azide	Interaction	ρ_b (au.)	$\nabla^2\rho_b$(au.)	ε	H_b(au.)	*DI*
**1a**	C^1-C^2	0.256	-0.618	0.033	-0.215	1.002
	C^2-C^3	0.339	-0.989	0.345	-0.385	1.780
	N^1-N^2	0.438	-0.911	0.251	-0.619	1.669
	N^2-N^3	0.573	-1.652	0.068	-1.010	2.417
	C^1-N^1	0.235	-0.534	0.020	-0.259	0.860
**1b**	C^1-C^2	0.340	-0.999	0.331	-0.388	1.834
	C^2-C^3	0.257	-0.625	0.026	-0.216	0.979
	N^1-N^2	0.573	-1.658	0.069	-1.013	2.419
	N^2-N^3	0.438	-0.911	0.248	-0.617	1.664
	C^3-N^3	0.232	-0.524	0.027	-0.253	0.834
**2a**	C^1-C^2	0.256	-0.618	0.031	-0.215	1.001
	C^2-C^3	0.339	-0.985	0.341	-0.385	1.777
	N^1-N^2	0.235	-0.534	0.019	-0.259	1.670
	N^2-N^3	0.572	-1.650	0.068	-1.010	2.416
	C^1-N^1	0.235	-0.534	0.019	-0.259	0.860
	$CH^2\cdots O$				0.000	0.037
**2b**	C^1-C^2	0.340	-1.000	0.329	-0.387	1.835
	C^2-C^3	0.257	-0.625	0.036	-0.216	0.984
	N^1-N^2	0.575	-1.669	0.069	-1.020	2.427
	N^2-N^3	0.437	-0.911	0.247	-0.614	1.654
	C^3-N^3	0.233	-0.521	0.010	-0.254	0.835
	$OH\cdots N^3$					0.023
**3a**	C^1-C^2	0.256	-0.620	0.028	-0.215	1.001
	C^2-C^3	0.339	-0.986	0.339	-0.385	1.769
	N^1-N^2	0.439	-0.916	0.250	-0.620	1.667
	N^2-N^3	0.572	-1.651	0.068	-1.009	2.410

	C^1-N^1	0.233	-0.528	0.017	-0.256	0.858
	$CH^2\cdots O$					0.037
	$CH\cdots N^3$	0.005	0.015	0.233	0.001	0.017
**3b**	C^1-C^2	0.339	-0.997	0.329	-0.387	1.833
	C^2-C^3	0.256	-0.622	0.028	-0.216	0.979
	N^1-N^2	0.573	-1.655	0.069	-1.013	2.415
	N^2-N^3	0.439	-0.916	0.247	-0.620	1.665
	C^3-N^3	0.234	-0.528	0.021	-0.254	0.836
	$CH^2\cdots O$					0.027
	$CH\cdots N^3$					0.015
**4a**	C^1-C^2	0.256	-0.619	0.032	-0.215	1.002
	C^2-C^3	0.336	-0.979	0.326	-0.379	1.741
	N^1-N^2	0.437	-0.906	0.252	-0.615	1.664
	N^2-N^3	0.573	-1.656	0.068	-1.012	2.420
	C^1-N^1	0.235	-0.537	0.018	-0.259	0.862
**4b**	C^1-C^2	0.337	-0.980	0.333	-0.381	1.832
	C^2-C^3	0.251	-0.594	0.024	-0.207	0.972
	N^1-N^2	0.557	-1.545	0.068	-0.954	2.409
	N^2-N^3	0.437	-0.905	0.240	-0.610	1.671
	C^3-N^3	0.231	-0.521	0.038	-0.245	0.843
	$CH\cdots N^3$					0.036

[a]See the text for an explanation of the symbols.

Atomic Charge

According to QTAIM, an atom is defined by the nucleus and its atomic basin, which is the volume enclosed by a zero-flux surface filled by the gradient lines of ρ(r) ending up at the nucleus.[8] In a molecule, each atomic basin is separated from their neighboring basins by a zero-flux surface in the gradient vector field of the charge density. The atomic electron population $N(\Omega)$ can be obtained by integrating the electron density over the atomic basin, which can be used to calculate the corresponding atomic net charge as $q(\Omega) = N(\Omega) - Z\Omega$, with $Z\Omega$ being the atomic number.

Table S3. Net charges for selected atoms (in e) in azides **1-4**.

Compound	$q(C^1)$	$q(C^2)$	$q(C^3)$	$q(N^1)$	$q(N^2)$	$q(N^3)$
**1a**	+0.330	-0.060	-0.035	-0.400	-0.164	+0.096
**1b**	-0.066	-0.055	+0.336	+0.101	-0.165	-0.400
**2a**	+0.332	-0.046	-0.051	-0.399	-0.164	+0.095
**2b**	-0.057	-0.057	+0.318	+0.117	-0.161	-0.415
**3a**	+0.329	-0.046	-0.057	-0.396	-0.165	+0.095
**3b**	-0.065	-0.051	+0.329	+0.098	-0.166	-0.393
**4a**	+0.334	-0.048	-0.038	-0.399	-0.164	+0.102
**4b**	-0.060	-0.052	+0.325	+0.090	-0.150	-0.391

Primary *vs* Tertiary azides

Table S4. Local topological properties for the selected bond critical point and delocalization indexes (*DI*) for azides **5-7**[a]

Azide	Interaction	ρ_b(au.)	$\nabla^2\rho_b$ (au.)	ε	H_b(au.)	*DI*
**5a**	C^1-C^2	0.254	-0.607	0.024	0.060	1.004
	C^2-C^3	0.334	-0.955	0.358	0.135	1.731
	N^1-N^2	0.440	-0.922	0.250	0.393	1.677
	N^2-N^3	0.571	-1.643	0.070	0.595	2.404
	C^1-N^1	0.232	-0.518	0.035	0.123	0.856
**5b**	C^1-C^2	0.339	-0.994	0.330	0.138	1.834
	C^2-C^3	0.253	-0.605	0.032	0.058	2.411
	N^1-N^2	0.573	-1.654	0.070	0.596	1.664
	N^2-N^3	0.438	-0.908	0.248	0.391	1.664
	C^3-N^3	0.228	-0.499	0.019	0.115	0.809
**6a**	C^1-C^2	0.254	-0.606	0.021	0.060	1.002
	C^2-C^3	0.334	-0.951	0.348	0.137	1.733
	N^1-N^2	0.441	-0.925	0.251	0.394	1.677
	N^2-N^3	0.571	-1.640	0.070	0.595	2.404
	C^1-N^1	0.232	-0.519	0.035	0.123	0.856
	$CH^2\cdots O$					0.043
**6b**	C^1-C^2	0.339	-0.992	0.328	0.138	1.832
	C^2-C^3	0.253	-0.604	0.030	0.059	0.965
	N^1-N^2	0.574	-1.665	0.071	0.600	2.417
	N^2-N^3	0.438	-0.915	0.246	0.388	1.656
	C^3-N^3	0.227	-0.488	0.011	0.116	0.809
	$CH^2\cdots O$					0.031
	$OH\cdots N^3$					0.022
**7a**	C^1-C^2	0.254	-0.608	0.023	0.060	1.002
	C^2-C^3	0.332	-0.946	0.346	0.133	1.707
	N^1-N^2	0.439	-0.915	0.252	0.391	1.671
	N^2-N^3	0.572	-1.647	0.070	0.596	2.409
	C^1-N^1	0.233	-0.522	0.032	0.123	0.859
**7b**	C^1-C^2	0.339	-0.994	0.331	0.138	1.824
	C^2-C^3	0.251	-0.593	0.029	0.058	0.957
	N^1-N^2	0.573	-1.657	0.070	0.599	2.413
	N^2-N^3	0.439	-0.917	0.245	0.390	1.659
	C^3-N^3	0.231	-0.518	0.032	0.114	0.819
	$CH\cdots N^3$	0.016	0.076	2.864	0.016	0.044

[a]See the text for an explanation of the symbols.

Table S5. Net charges for selected atoms (in e) in azides **5-7**.

Compound	$q(C^1)$	$q(C^2)$	$q(C^3)$	$q(N^1)$	$q(N^2)$	$q(N^3)$
**5a**	0.320	-0.065	-0.017	-0.395	-0.165	0.088
**5b**	-0.069	-0.058	0.329	0.096	-0.168	-0.391
**6a**	0.324	-0.063	-0.041	-0.393	-0.165	0.088
**6b**	-0.066	-0.058	0.309	0.110	-0.165	-0.403
**7a**	0.323	-0.052	-0.022	-0.393	-0.165	0.096
**7b**	-0.063	-0.054	0.326	0.106	-0.167	-0.390

Secondary *vs* Tertiary azides

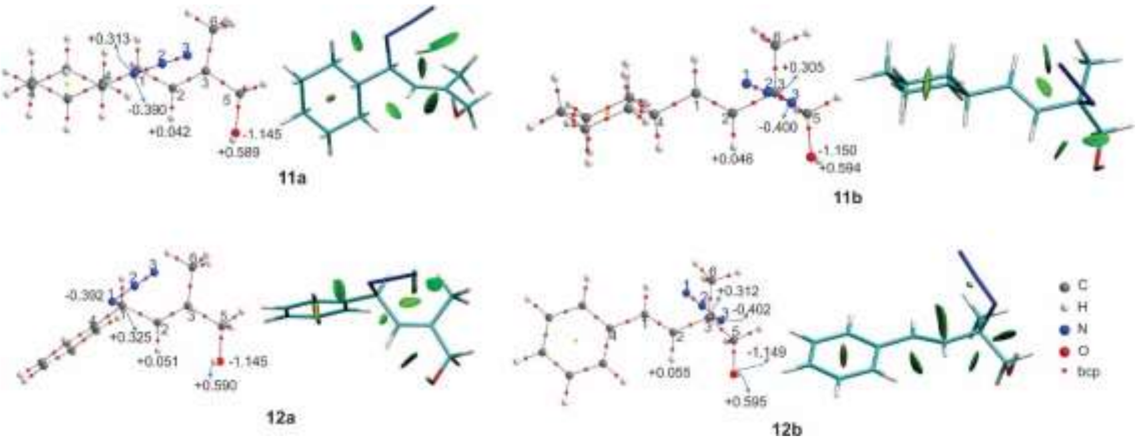

Figure S4. Molecular graphs of the azides **11** and **12** (left). For selected atoms, the atomic charges $q(\Omega)$ are given in *e*. NCI gradient isosurfaces (right), represented at an isovalue of 0.5 a.u. and blue-green-red color scale from $-0.05 < \rho < +0.05$ a.u.

Table S6. Local topological properties for the selected bond critical point and delocalization indexes (*DI*) for azides **8-12**[a]

Azide	Interaction	ρ_b(au.)	$\nabla^2\rho_b$ (au.)	ε	H_b(au.)	*DI*
**8a**	C^1-C^2	0.255	-0.610	0.023	-0.213	0.985
	C^2-C^3	0.334	-0.955	0.359	-0.374	1.730
	N^1-N^2	0.441	-0.925	0.246	-0.625	1.676
	N^2-N^3	0.571	-1.646	0.070	-1.007	2.404
	C^1-N^1	0.228	-0.501	0.039	-0.244	0.827
**8b**	C^1-C^2	0.338	-0.984	0.345	-0.384	1.778
	C^2-C^3	0.253	-0.601	0.035	-0.209	0.966
	N^1-N^2	0.572	-1.651	0.070	-1.008	2.408
	N^2-N^3	0.439	-0.914	0.247	-0.622	1.668
	C^3-N^3	0.226	-0.491	0.021	-0.237	0.805
**9a**	C^1-C^2	0.255	-0.609	0.021	-0.213	0.988
	C^2-C^3	0.334	-0.954	0.357	-0.374	1.729
	N^1-N^2	0.440	-0.925	0.243	-0.621	1.667
	N^2-N^3	0.573	-1.656	0.071	-1.014	2.409
	C^1-N^1	0.228	-0.492	0.038	-0.243	0.829
	$OH\cdots N^1$					0.021
**9b**	C^1-C^2	0.338	-0.979	0.341	-0.384	1.775
	C^2-C^3	0.253	-0.601	0.033	-0.209	0.965
	N^1-N^2	0.572	-1.649	0.070	-1.008	2.408
	N^2-N^3	0.439	-0.916	0.247	-0.622	1.669
	C^3-N^3	0.226	-0.492	0.020	-0.237	0.805
**10a**	C^1-C^2	0.255	-0.610	0.021	-0.213	0.984
	C^2-C^3	0.334	-0.950	0.349	-0.375	1.732
	N^1-N^2	0.441	-0.929	0.246	-0.627	1.676
	N^2-N^3	0.571	-1.642	0.070	-1.005	2.404
	C^1-N^1	0.228	-0.501	0.039	-0.244	0.827
	C^3-N^3					0.012
**10b**	C^1-C^2	0.338	-0.981	0.343	-0.383	1.775
	C^2-C^3	0.253	-0.601	0.032	-0.210	0.969
	N^1-N^2	0.574	-1.662	0.071	-1.015	2.414

	N^2-N^3	0.439	-0.920	0.245	-0.620	1.661
	C^3-N^3	0.225	-0.479	0.014	-0.235	0.805
	OH···N^3					0.023
**11a**	C^1-C^2	0.254	-0.606	0.022	-0.212	0.981
	C^2-C^3	0.334	-0.950	0.351	-0.375	1.725
	N^1-N^2	0.443	-0.935	0.241	-0.630	1.679
	N^2-N^3	0.570	-1.638	0.069	-1.005	2.400
	C^1-N^1	0.227	-0.495	0.031	-0.243	0.823
**11b**	C^1-C^2	0.337	-0.979	0.336	-0.382	1.764
	C^2-C^3	0.253	-0.601	0.030	-0.210	0.970
	N^1-N^2	0.574	-1.661	0.071	-1.014	2.413
	N^2-N^3	0.439	-0.921	0.244	-0.621	1.661
	C^3-N^3	0.224	-0.475	0.015	-0.233	0.804
	OH···N^3					0.023
**12a**	C^1-C^2	0.252	-0.593	0.022	-0.209	0.976
	C^2-C^3	0.334	-0.949	0.351	-0.374	1.726
	N^1-N^2	0.442	-0.934	0.245	-0.627	1.668
	N^2-N^3	0.572	-1.647	0.069	-1.009	2.410
	C^1-N^1	0.234	-0.530	0.048	-0.252	0.839
**12b**	C^1-C^2	0.336	-0.973	0.322	-0.378	1.737
	C^2-C^3	0.254	-0.602	0.034	-0.210	0.970
	N^1-N^2	0.575	-1.667	0.071	-1.017	2.418
	N^2-N^3	0.438	-0.913	0.247	-0.616	1.656
	C^3-N^3	0.226	-0.485	0.014	-0.236	0.807
	OH···N^3					0.023

[a]See the text for an explanation of the symbols.

Table S7. Atomic Net Charge (in *e*) of selected atoms in azides **8-12**.

Compounds	$q(C^1)$	$q(C^2)$	$q(C^3)$	$q(N^1)$	$q(N^2)$	$q(N^3)$	$q(O)$	$q(H)$
**10a**	0.325	-0.071	-0.018	-0.394	-0.167	0.088		
**10b**	-0.039	-0.069	0.327	0.090	-0.169	-0.390		
**11a**	0.304	-0.073	-0.015	-0.406	-0.163	0.102	-1.148	+0.593
**11b**	-0.055	-0.056	0.330	0.089	-0.169	-0.389	-1.148	+0.595
**12a**	0.328	-0.070	-0.041	-0.392	-0.167	0.087	-1.145	+0.589
**12b**	-0.036	-0.069	0.307	0.104	-0.166	-0.402	-1.150	+0.594
**13a**	0.313	-0.067	-0.041	-0.390	-0.168	0.083	-1.145	+0.589
**13b**	-0.048	-0.073	0.305	0.103	-0.166	-0.400	-1.150	+0.594
**14a**	0.325	-0.067	-0.036	-0.392	-0.164	0.099	-1.145	+0.590
**14b**	-0.038	-0.056	0.312	0.111	-0.166	-0.402	-1.149	+0.595

Cartesian**¡Error! Marcador no definido.** coordinates, thermal correction free energies (calculated at M06-2X/6-31+G(d,p)) and absolute electronic energies (calculated at M06-2X/6-311++G(d,p) in chloroform) of azides under study.

1a

C	0.524501	-1.320336	0.267571
C	-0.724507	-0.726439	-0.312266
C	-1.589887	-0.000643	0.398177
C	-2.831985	0.633367	-0.151021
N	1.755869	-0.684794	-0.276683
N	1.801795	0.529541	-0.064942
N	1.919858	1.644407	0.083535
H	0.628544	-2.374331	0.005865
H	0.516097	-1.238007	1.362253
H	-0.886605	-0.879761	-1.379105
H	-1.396318	0.146371	1.462412
H	-3.722437	0.250805	0.358654
H	-2.814261	1.717096	0.002915
H	-2.936405	0.438059	-1.221122

Thermal correction to Gibbs Free Energy= 0.079961 au.
Absolute Electronic Energy = -320.752348 au.

1b

C	-1.814761	-1.325383	-0.068990
C	-0.654561	-0.367735	-0.290099
C	-0.840993	0.940232	0.426992
C	-0.709196	2.128626	-0.159188
N	0.567427	-1.064967	0.215508
N	1.601884	-0.414686	0.043099
N	2.591486	0.118765	-0.076108
H	-2.739864	-0.885985	-0.449619
H	-1.629031	-2.272259	-0.581083
H	-1.933960	-1.528738	0.999175
H	-0.528671	-0.174362	-1.365147
H	-1.066676	0.867169	1.490981
H	-0.475722	2.212398	-1.218238
H	-0.834584	3.053548	0.394145

Thermal correction to Gibbs Free Energy= 0.080605 au.
Absolute Electronic Energy = -320.7512316 au.

2a

C	1.054106	-1.350406	0.205815
C	-0.297075	-0.788777	-0.121950
C	-1.056242	-0.165519	0.778338
C	-2.394603	0.439782	0.484699
O	-2.725553	0.222528	-0.870699
N	2.151156	-0.595834	-0.460185
N	2.168430	0.597435	-0.149524
N	2.252386	1.701562	0.081046
H	1.164912	-2.369849	-0.165977
H	1.217705	-1.360443	1.291483
H	-0.639255	-0.865815	-1.151171
H	-0.717812	-0.073022	1.810982
H	-3.144630	-0.011193	1.152311
H	-2.354339	1.515058	0.715697
H	-3.563083	0.652414	-1.068498

Thermal correction to Gibbs Free Energy= 0.08379 au.
Absolute Electronic Energy = -395.9704248 au.

2b

C	-1.823282	0.364700	0.193184
C	-0.343982	0.296465	-0.174480
C	0.448450	1.424876	0.416785
C	1.234040	2.225876	-0.300854
O	-2.544076	-0.715457	-0.345714
N	0.117663	-1.019904	0.359464
N	1.287421	-1.285952	0.067890
N	2.351965	-1.590697	-0.153280
H	-1.913465	0.401408	1.289215
H	-2.252859	1.280242	-0.220975
H	-0.248898	0.292498	-1.269343
H	0.367664	1.549816	1.496496
H	1.332026	2.105142	-1.377208
H	1.799214	3.028491	0.161550
H	-2.121778	-1.529573	-0.040355

Thermal correction to Gibbs Free Energy= 0.08517 au.
Absolute Electronic Energy = -395.9718417 au.

3a

C	0.941899	1.557490	-0.654307
C	1.433381	1.136959	0.510417
C	-0.526756	1.692791	-0.949782
C	2.895779	0.925742	0.759893
O	-1.334573	1.247528	0.111197
N	2.796832	-1.719374	-1.164836
N	2.989066	-1.113843	-0.228940
N	3.226138	-0.526933	0.829453
Si	-1.988653	-0.301177	0.144427
C	-2.767434	-0.451398	1.834575
C	-3.271466	-0.456405	-1.218279
C	-0.642967	-1.583898	-0.118761

H	1.617040	1.834392	-1.465889
H	0.748180	0.867970	1.311749
H	-0.763837	2.747628	-1.141447
H	-0.748971	1.151540	-1.885272
H	3.205046	1.321062	1.727962
H	3.499974	1.406527	-0.020233
H	-3.533399	0.316363	1.977941
H	-3.236316	-1.431650	1.966913
H	-2.013633	-0.326926	2.618056
H	-3.748322	-1.441951	-1.185054
H	-4.052556	0.301866	-1.106796
H	-2.826153	-0.341437	-2.212059
H	-1.079994	-2.586805	-0.178680
H	-0.093620	-1.408302	-1.050364
H	0.085413	-1.580654	0.699346

Thermal correction to Gibbs Free Energy= 0.177375 au.
Absolute Electronic Energy = -804.6417713 au.

3b

C	-1.544127	-0.493064	-0.678440
C	-2.087940	-1.294336	0.469736
C	-0.120952	-0.895902	-1.046762
C	-3.296648	-1.854091	0.457430
O	0.708867	-0.836527	0.086059
N	-1.484890	0.959248	-0.340617
N	-2.574721	1.425589	-0.001579
N	-3.535855	1.925918	0.321310
Si	2.114592	0.082298	0.144101
C	2.779467	-0.177496	1.869970
C	3.310960	-0.559155	-1.155510
C	1.733980	1.887201	-0.183972
H	-2.184117	-0.628172	-1.563426
H	-1.437458	-1.366462	1.338574
H	-0.138890	-1.923072	-1.432021
H	0.226871	-0.230122	-1.851143
H	-3.671653	-2.411308	1.309861
H	-3.949047	-1.778160	-0.409867
H	2.964217	-1.238973	2.059585
H	3.719788	0.364049	2.014683
H	2.062802	0.180840	2.615061
H	4.250513	0.002942	-1.130094
H	3.542721	-1.615356	-0.986385
H	2.897005	-0.460639	-2.164839
H	2.652989	2.482646	-0.151103
H	1.272614	2.031563	-1.166132
H	1.036244	2.276053	0.562842

Thermal correction to Gibbs Free Energy= 0.175932 au.
Absolute Electronic Energy = -804.6392675 au.

4a

C	-2.632929	0.808224	-0.975776
C	-1.226441	0.289805	-0.971903
C	-0.278449	0.776212	-0.162604
C	1.118963	0.317554	-0.065720
N	-3.601920	-0.197065	-0.460313
N	-3.375198	-0.524170	0.708363
N	-3.236893	-0.879994	1.772489
C	2.051929	1.127769	0.592698
C	3.386832	0.745066	0.697103
C	3.809028	-0.462933	0.147601
C	2.886216	-1.285995	-0.500102
C	1.554291	-0.902132	-0.603535
H	-2.703109	1.732451	-0.387596
H	-2.978648	1.020074	-1.988624
H	-1.017857	-0.536360	-1.649069
H	-0.536674	1.616955	0.483403
H	1.724507	2.071162	1.022512
H	4.095050	1.389532	1.208437
H	4.847713	-0.767319	0.228645
H	3.206371	-2.235458	-0.918026
H	0.844082	-1.563856	-1.090028

Thermal correction to Gibbs Free Energy= 0.130419 au.
Absolute Electronic Energy = -512.4701186 au.

4b

C	-0.686668	0.006432	-0.157906
C	0.815996	0.227465	-0.247208
C	1.306798	1.195490	0.805581
C	2.003339	2.302382	0.534859
N	1.497697	-1.098544	-0.094945
N	2.716485	-1.085015	-0.291192
N	3.842276	-1.190081	-0.452290
C	-1.238006	-1.096221	0.504691
C	-2.625399	-1.236156	0.606870
C	-3.474783	-0.278844	0.047919
C	-2.929080	0.823263	-0.617220
C	-1.544136	0.963719	-0.717806
H	1.056401	0.632349	-1.239893
H	1.064125	0.929511	1.833275
H	2.255304	2.585397	-0.484829
H	2.341038	2.966698	1.324563
H	-0.580841	-1.849545	0.925613
H	-3.040445	-2.098934	1.120079
H	-4.552255	-0.391574	0.125207
H	-3.580291	1.571098	-1.060509
H	-1.126608	1.825303	-1.233199

Thermal correction to Gibbs Free Energy= 0.127804 au.

Absolute Electronic Energy = -512.4615727 au.

5a

C	0.846099	-1.346774	0.468254
C	-0.423691	-1.058719	-0.278812
C	-1.325885	-0.118587	0.037732
C	-1.237768	0.790903	1.235692
C	-2.540288	0.106597	-0.823510
N	2.026972	-0.636579	-0.107638
N	1.830209	0.566964	-0.280112
N	1.723993	1.675636	-0.481569
H	1.108688	-2.402294	0.396844
H	0.765113	-1.098432	1.531897
H	-0.577366	-1.635478	-1.189270
H	-0.361011	0.609603	1.859049
H	-1.202950	1.835422	0.905935
H	-2.132077	0.682063	1.859822
H	-3.456971	-0.046017	-0.242013
H	-2.563154	1.139126	-1.189986
H	-2.559282	-0.566663	-1.683187

Thermal correction to Gibbs Free Energy= 0.106702 au.
Absolute Electronic Energy = -360.0607483 au.

5b

C	0.577378	-0.365020	0.001141
C	0.705288	0.985839	-0.666241
C	1.673074	-1.299396	-0.506108
C	0.753422	2.166510	-0.052450
C	0.584577	-0.299744	1.526669
N	-0.705717	-0.998302	-0.470416
N	-1.709688	-0.341309	-0.184313
N	-2.675932	0.202412	0.039681
H	0.732359	0.942843	-1.755312
H	1.639453	-1.369555	-1.596551
H	2.653102	-0.919876	-0.204868
H	1.531780	-2.301061	-0.091141
H	0.830255	3.086545	-0.622582
H	0.723589	2.260800	1.029063
H	0.465991	-1.306901	1.934055
H	1.530791	0.114075	1.886978
H	-0.230398	0.324395	1.907620

Thermal correction to Gibbs Free Energy= 0.108023au.
Absolute Electronic Energy = -360.0597569 au.

6a

C	0.552330	1.676722	-0.899485
C	0.937951	0.300783	-0.425461
C	2.302215	0.255229	0.229160
C	0.198037	-0.808330	-0.548937
C	-1.183553	-0.877877	-1.131860
N	-2.031977	0.938195	1.471620
N	-2.103877	0.112015	0.701075
N	-2.247048	-0.824892	-0.084886
O	2.631029	-0.969263	0.841605
H	1.334645	2.089762	-1.546826
H	0.448738	2.356173	-0.046040
H	-0.386833	1.692914	-1.453525
H	3.068791	0.426943	-0.535644
H	2.377627	1.089700	0.943581
H	0.602141	-1.740071	-0.160518
H	-1.367100	-0.086349	-1.865989
H	-1.346152	-1.833750	-1.629616
H	2.058345	-1.097600	1.606568

Thermal correction to Gibbs Free Energy= 0.110916 au.
Absolute Electronic Energy = -435.2795194au.

6b

C	-0.102635	0.620303	1.719421
C	0.352919	0.137121	0.347422
C	1.841656	-0.237267	0.386516
C	0.091125	1.110709	-0.776987
C	-0.675269	2.197197	-0.698206
N	-0.313375	-1.174470	0.017447
N	-1.531009	-1.077386	-0.154457
N	-2.648504	-1.055391	-0.319803
O	2.285869	-0.795627	-0.825018
H	0.411946	1.547952	1.986569
H	0.122056	-0.139736	2.472205
H	-1.180249	0.809616	1.739447
H	2.431727	0.667810	0.558113
H	2.001948	-0.929081	1.226068
H	0.571654	0.845017	-1.716867
H	-1.171156	2.497584	0.220419
H	-0.825193	2.833471	-1.564387
H	1.753745	-1.585263	-0.992724

Thermal correction to Gibbs Free Energy= 0.112037 au.
Absolute Electronic Energy = -435.2807356 au.

7a

C	-2.570473	0.281590	-1.289494
C	-1.112144	-0.008425	-1.084513
C	-0.285174	0.644772	-0.250392
C	1.130117	0.198846	-0.111224
C	-0.687605	1.848439	0.568170

N	-3.447968	-0.627987	-0.495690
N	-3.168237	-0.642572	0.705057
N	-2.969936	-0.721470	1.816163
C	1.465462	-1.161857	-0.139039
C	2.790029	-1.573310	-0.025835
C	3.807627	-0.632208	0.124565
C	3.486588	0.722699	0.170815
C	2.160140	1.132625	0.062235
H	-2.857900	0.092908	-2.323917
H	-2.825107	1.321440	-1.060855
H	-0.721576	-0.853824	-1.646881
H	-0.334984	1.737155	1.597725
H	-0.239151	2.763519	0.166003
H	-1.767565	1.996140	0.596014
H	0.676222	-1.903129	-0.223112
H	3.026363	-2.632857	-0.041633
H	4.840342	-0.953608	0.215638
H	4.269724	1.464535	0.292917
H	1.929210	2.192899	0.097651

Thermal correction to Gibbs Free Energy= 0.155387 au.
Absolute Electronic Energy = -551.7757306 au.

7b

C	-1.066506	0.404783	1.633481
C	-0.721661	0.122364	0.167078
C	0.789271	-0.032064	0.021106
C	-1.232174	1.176140	-0.794335
C	-1.996827	2.218770	-0.477391
N	-1.327271	-1.190377	-0.238779
N	-2.558794	-1.193317	-0.191999
N	-3.686480	-1.274498	-0.178437
C	1.398170	-1.281311	-0.102847
C	1.590100	1.114041	0.067102
C	2.976377	1.013227	-0.005541
C	3.580779	-0.237577	-0.127528
C	2.787724	-1.380854	-0.176330
H	-0.611317	1.344253	1.957790
H	-0.672798	-0.403546	2.253893
H	-2.149404	0.470291	1.783567
H	-0.948501	1.001640	-1.831579
H	-2.300332	2.427958	0.544206
H	-2.341273	2.906671	-1.242608
H	0.782378	-2.172480	-0.147185
H	1.122859	2.092250	0.153680
H	3.584062	1.912228	0.027791
H	4.661633	-0.318361	-0.187701
H	3.248993	-2.358666	-0.276130

Thermal correction to Gibbs Free Energy= 0.15581 au.
Absolute Electronic Energy = -551.7703333 au.

8a

C	-1.046287	0.695113	0.320582
C	0.275107	0.826706	-0.384417
C	1.452160	0.330666	0.021739
C	1.672628	-0.437094	1.299608
C	2.693440	0.511524	-0.811870
N	-1.837337	-0.458550	-0.226586
N	-1.201855	-1.512789	-0.257469
N	-0.676871	-2.513083	-0.326096
C	-1.920353	1.925881	0.140543
H	-0.897707	0.517974	1.392435
H	0.232687	1.343168	-1.344008
H	2.074060	-1.430582	1.070034
H	2.416670	0.072356	1.922699
H	0.766735	-0.574265	1.891587
H	2.490238	1.075210	-1.724998
H	3.466234	1.039902	-0.241623
H	3.112129	-0.462268	-1.090394
H	-2.098775	2.107869	-0.923236
H	-2.885505	1.784385	0.632177
H	-1.424500	2.800424	0.569273

Thermal correction to Gibbs Free Energy= 0.133164 au.
Absolute Electronic Energy = -399.3694579 au.

8b

C	-1.031654	-2.038945	-0.648011
C	-0.599867	-0.718256	-0.015511
C	-0.780399	-0.765505	1.500898
C	0.802499	-0.368762	-0.456332
C	1.821726	-0.025077	0.332051
N	-1.547409	0.302744	-0.596465
N	-1.297292	1.455242	-0.238604
N	-1.126688	2.537229	0.045199
C	3.191808	0.330309	-0.165146
H	-0.404211	-2.851856	-0.272624
H	-2.076876	-2.248325	-0.404076
H	-0.934329	-1.990089	-1.735843
H	-0.545739	0.196867	1.967564
H	-0.129766	-1.527619	1.939511
H	-1.818603	-1.015264	1.733979
H	0.948537	-0.386421	-1.538075
H	1.680438	0.000497	1.412119
H	3.469216	1.341515	0.149786
H	3.242279	0.283225	-1.255871
H	3.944092	-0.351618	0.244922

Thermal correction to Gibbs F ree Energy= 0.133988au.
Absolute Electronic Energy = -399.3693755 au.

9a

C	-1.555577	-0.684531	0.036747
C	-0.236963	-0.804045	-0.172784
C	-2.544598	-1.395037	-0.848930
C	0.844657	-0.138691	0.629104
C	-2.183086	0.137361	1.133129
N	-0.511536	2.599044	-0.640121
N	0.305421	1.897660	-0.296067
N	1.250419	1.186751	0.049291
C	2.137582	-0.951528	0.639874
O	2.650786	-1.133045	-0.659258
H	0.116883	-1.401885	-1.011305
H	-3.219784	-0.674505	-1.324349
H	-2.046188	-1.972483	-1.630255
H	-3.168944	-2.075060	-0.257742
H	0.534210	0.025100	1.668511
H	-1.458434	0.659338	1.760550
H	-2.850029	0.890413	0.698360
H	-2.799071	-0.500200	1.777672
H	2.868508	-0.450006	1.288829
H	1.933693	-1.946529	1.044451
H	2.800655	-0.255175	-1.035210

Thermal correction to Gibbs Free Energy= 0.137661 au.
Absolute Electronic Energy = -474.5904045 au.

9b

C	-1.453698	-0.766468	1.419703
C	-1.001341	-0.699000	-0.037843
C	-1.378419	-1.979694	-0.778043
C	0.473066	-0.419710	-0.213743
C	1.335111	-0.131972	0.759756
N	-1.775425	0.386292	-0.745009
N	-1.520165	1.516218	-0.324970
N	-1.335579	2.581943	0.007826
C	2.787259	0.164329	0.532974
O	3.091510	0.052102	-0.841550
H	-1.256208	0.171942	1.948579
H	-0.933273	-1.573503	1.943657
H	-2.528606	-0.960526	1.457882
H	-0.874414	-2.834457	-0.318953
H	-2.460249	-2.133414	-0.735424
H	-1.078284	-1.914284	-1.827044
H	0.830000	-0.433849	-1.242414
H	1.019788	-0.101794	1.801506
H	3.392058	-0.536856	1.128311
H	3.001703	1.178050	0.903915
H	4.001722	0.325789	-0.989360

Thermal correction to Gibbs Free Energy= 0.138009 au.
Absolute Electronic Energy = -474.5876766 au.

10a

C	-1.132720	0.012590	0.517903
C	-0.156149	-0.709727	-0.044241
C	-2.537958	-0.012965	-0.045797
C	1.286301	-0.744014	0.376224
O	-2.676415	-0.677737	-1.279329
C	-0.997311	0.902528	1.725209
N	1.286745	2.370975	-0.683648
N	1.656115	1.307873	-0.561199
N	2.111178	0.166523	-0.488053
C	1.895338	-2.129507	0.232401
H	-0.401854	-1.314611	-0.916215
H	-3.194248	-0.539933	0.657110
H	-2.909644	1.021809	-0.108337
H	1.400403	-0.407836	1.413699
H	-2.200546	-0.180428	-1.954782
H	-0.026668	0.819752	2.215031
H	-1.142423	1.951192	1.441731
H	-1.769874	0.659885	2.464160
H	1.367512	-2.836147	0.877691
H	1.813169	-2.470317	-0.803602
H	2.952214	-2.112497	0.508252

Thermal correction to Gibbs Free Energy= 0.136751 au.
Absolute Electronic Energy = -474.5882512 au.

10b

C	-0.591986	-0.208002	0.417381
C	0.708459	-0.626367	-0.222802
C	-1.651749	-1.303424	0.229233
C	1.925553	-0.462721	0.298629
O	-1.875050	-1.605608	-1.126106
C	-0.483921	0.166423	1.891549
N	-1.174151	0.951053	-0.357098
N	-0.412404	1.916423	-0.440150
N	0.239801	2.833129	-0.548478
C	3.195905	-0.872319	-0.385839
H	0.597416	-1.076414	-1.209116
H	-1.302406	-2.222186	0.709375
H	-2.579365	-0.976674	0.721250
H	2.036758	-0.013199	1.285149
H	-2.152571	-0.787777	-1.560804
H	0.196862	1.010099	2.040889

H	-1.467310	0.453630	2.272811
H	-0.110405	-0.681164	2.473778
H	3.748376	-1.598456	0.219672
H	2.993050	-1.319696	-1.361895
H	3.853701	-0.009083	-0.531070

Thermal correction to Gibbs Free Energy= 0.138507au.
Absolute Electronic Energy = -474.5905088 au.

11a

C	-2.250696	-0.678623	0.477679
C	-1.152525	-0.394822	-0.232183
C	-3.309133	-1.613688	-0.068792
C	-0.063928	0.568271	0.150710
O	-3.136979	-1.993437	-1.413896
C	-2.584102	-0.147512	1.846830
C	1.325606	0.099146	-0.282784
C	2.400761	1.141596	0.041877
C	1.667313	-1.245464	0.372564
C	3.063482	-1.728905	-0.028031
C	4.131457	-0.681920	0.293866
C	3.794454	0.657673	-0.363418
N	-2.467483	2.789503	-0.202992
N	-1.437392	2.334960	-0.322824
N	-0.303768	1.896954	-0.509668
H	-1.040286	-0.856291	-1.213166
H	-4.297467	-1.154939	0.090775
H	-3.297606	-2.543549	0.512065
H	-0.044003	0.735044	1.236925
H	-3.259278	-1.217186	-1.972819
H	-2.851100	-0.971812	2.518342
H	-1.763805	0.405843	2.304949
H	-3.452387	0.519244	1.795041
H	1.296584	-0.041046	-1.375536
H	2.385112	1.334694	1.126147
H	2.162508	2.087079	-0.453602
H	0.915258	-1.998185	0.110867
H	1.625060	-1.124047	1.466369
H	3.074753	-1.935161	-1.107468
H	3.288681	-2.675013	0.476030
H	5.117209	-1.031784	-0.031742
H	4.185981	-0.545251	1.383317
H	4.543571	1.412603	-0.100902
H	3.831404	0.542818	-1.455952

Thermal correction to Gibbs Free Energy= 0.254064 au.
Absolute Electronic Energy = -669.9190227 au.

11b

C	-2.317273	-0.361091	0.327335
C	-0.919638	-0.474321	-0.229393
C	-3.190787	-1.515185	-0.183544
C	0.208094	-0.205866	0.431614
O	-3.252862	-1.560727	-1.588007
C	-2.391357	-0.284831	1.848699
C	1.584407	-0.273367	-0.163981
C	2.226093	1.124525	-0.198888
C	2.476963	-1.248946	0.620536
C	3.900313	-1.292369	0.059564
C	4.525794	0.103711	0.028161
C	3.649023	1.078919	-0.760531
N	-2.989499	0.854092	-0.271059
N	-2.355809	1.896300	-0.094720
N	-1.816410	2.880711	0.038294
H	-0.876254	-0.767324	-1.278344
H	-4.193635	-1.411129	0.255109
H	-2.757489	-2.462897	0.149318
H	0.170872	0.092222	1.482137
H	-3.600705	-0.709898	-1.888133
H	-1.941431	-1.175767	2.296538
H	-1.863380	0.593534	2.232149
H	-3.435679	-0.215433	2.164282
H	1.499336	-0.637970	-1.198772
H	2.251184	1.526580	0.825253
H	1.596795	1.800811	-0.788252
H	2.026783	-2.248200	0.610459
H	2.511470	-0.925234	1.671893
H	3.872655	-1.698721	-0.961204
H	4.516919	-1.974825	0.654556
H	5.531626	0.060382	-0.403626
H	4.637183	0.472027	1.057859
H	4.087936	2.082556	-0.750522
H	3.610940	0.760305	-1.811678

Thermal correction to Gibbs Free Energy= 0.254822 au.
Absolute Electronic Energy = -669.9214287 au.

12a

C	2.066210	0.782318	0.357519
C	0.920333	0.360893	-0.190743
C	2.979654	1.727370	-0.395294
C	-0.050861	-0.617166	0.417805
C	2.591714	0.417113	1.720656
N	2.333378	-2.806418	-0.107249
N	1.286778	-2.376819	-0.120815
N	0.125834	-1.973723	-0.183048
O	2.635279	1.948526	-1.741787
C	-1.487112	-0.180689	0.207253
C	-1.906617	1.006984	0.814748
C	-3.209928	1.463418	0.649526

C	-4.110018	0.736760	-0.131087
C	-3.694860	-0.443714	-0.739894
C	-2.387518	-0.903969	-0.572527
H	0.654476	0.717556	-1.184150
H	2.950742	2.710512	0.089613
H	4.014212	1.360882	-0.303412
H	0.119275	-0.707343	1.498023
H	3.491593	-0.201437	1.627846
H	2.879544	1.321274	2.269357
H	1.871972	-0.129226	2.331005
H	2.754284	1.127633	-2.233616
H	-1.201951	1.578354	1.415248
H	-3.523497	2.386399	1.127487
H	-5.127362	1.091757	-0.262395
H	-4.388693	-1.014932	-1.349082
H	-2.064734	-1.826814	-1.041619

Thermal correction to Gibbs Free Energy= 0.183625 au.
Absolute Electronic Energy = -666.2997759 au.

12b

C	2.098203	0.132618	0.462161
C	0.653532	0.372173	0.103293
C	2.819954	1.475618	0.654085
C	-0.394600	-0.270254	0.630557
C	2.315997	-0.755993	1.681621
N	2.810840	-0.456944	-0.730372
N	2.309658	-1.511063	-1.129950
N	1.900687	-2.480378	-1.541546
O	2.743435	2.291703	-0.488629
C	-1.812196	-0.051981	0.284745
C	-2.769234	-0.950646	0.772503
C	-4.119853	-0.797326	0.469173
C	-4.537591	0.266360	-0.326454
C	-3.594967	1.175290	-0.811025
C	-2.247398	1.020339	-0.507545
H	0.514230	1.126265	-0.668677
H	2.341888	2.022204	1.471912
H	3.865019	1.269643	0.926740
H	-0.227134	-1.034573	1.388390
H	3.387173	-0.871349	1.865932
H	1.849357	-0.313316	2.566558
H	1.889210	-1.752633	1.531933
H	3.138920	1.800105	-1.221403
H	-2.447371	-1.783459	1.392826
H	-4.843837	-1.507993	0.855387
H	-5.589191	0.392566	-0.563727
H	-3.913372	2.013298	-1.423337
H	-1.531742	1.747110	-0.879114

Thermal correction to Gibbs Free Energy= 0.186581 au.
Absolute Electronic Energy = -666.3084961 au.

References

1. Y. Zhao and D. Truhlar, *Theor. Chem. Acc.*, 2008, **120**, 215-241.
2. Y. Zhao and D. G. Truhlar, *J. Phys. Chem. A*, 2004, **108**, 6908-6918.
3. A. D. Becke, *J. Chem. Phys.*, 1993, **98**, 5648-5652.
4. C. Lee, W. Yang and R. G. Parr, *Phys. Rev. B*, 1988, **37**, 785-789.
5. A. V. Marenich, C. J. Cramer and D. G. Truhlar, *J. Phys. Chem. B*, 2009, **113**, 6378-6396.
6. S. Grimme, S. Ehrlich and L. Goerigk, *J. Comput. Chem*, 2011, **32**, 1456-1465.
7. A. K. Feldman, B. Colasson, K. B. Sharpless and V. V. Fokin, *J. Am. Chem. Soc.*, 2005, **127**, 13444-13445.
8. R. F. W. Bader, *Atoms in Molecules. A Quantum Theory*, Oxford Science Publications, Clarendon Press, London 1990.
9. C. F. Matta and R. J. Boyd, *The Quantum Theory of Atoms in Molecules: from solid state to DNA and drug design*, Wiley-VCH, Weinheim, 2007.
10. D. Cremer and E. Kraka, *Angew. Chem. Int. Ed. Eng.*, 1984, **23**, 627-628.
11. C. S. López, O. N. Faza, F. P. Cossío, D. M. York and A. R. de Lera, *Chem. Eur. J.*, 2005, **11**, 1734-1738.
